# Natural Medicines Used in the Traditional Tibetan Medical System for the Treatment of Liver Diseases

**DOI:** 10.3389/fphar.2018.00029

**Published:** 2018-01-30

**Authors:** Qi Li, Hai-Jiao Li, Tong Xu, Huan Du, Chen-Lei Huan Gang, Gang Fan, Yi Zhang

**Affiliations:** ^1^College of Pharmacy, Chengdu University of Traditional Chinese Medicine, Chengdu, China; ^2^College of Ethnic Medicine, Chengdu University of Traditional Chinese Medicine, Chengdu, China; ^3^Characteristic National Medicine Innovation Research Center of Tibet-Qiang-Yi, Chengdu University of Traditional Chinese Medicine, Chengdu, China

**Keywords:** traditional Tibetan medicine, natural medicines, liver diseases, hepatitis, *Carthamus tinctorius*, Brag-zhun, *Swertia chirayita*

## Abstract

Liver disease is one of the most risk factors threatening human health. It is of great significance to find drugs that can treat liver diseases, especially for acute and chronic hepatitis, non-alcoholic fatty liver disease, and liver cancer. The search for drugs with good efficacy from traditional natural medicines has attracted more and more attention. Tibetan medicine, one of the China's traditional medical systems, has been widely used by the Tibetan people for the prevention and treatment of liver diseases for hundreds of years. The present paper summarized the natural Tibetan medicines that have been used in Tibetan traditional system of medicine to treat liver diseases by bibliographic investigation of 22 Tibetan medicine monographs and drug standards. One hundred and ninety three species including 181 plants, 7 animals, and 5 minerals were found to treat liver diseases in traditional Tibetan medicine system. The most frequently used species are *Carthamus tinctorius*, Brag-zhun, *Swertia chirayita, Swertia mussotii, Halenia elliptica, Herpetospermum pedunculosum*, and *Phyllanthus emblica*. Their names, families, medicinal parts, traditional uses, phytochemicals information, and pharmacological activities were described in detail. These natural medicines might be a valuable gift from the old Tibetan medicine to the world, and would be potential drug candidates for the treatment of liver diseases. Further studies are needed to prove their medicinal values in liver diseases treatment, identify bioactive compounds, elucidate the underlying mechanism of action, and clarify their side effects or toxicity with the help of modern phytochemical, pharmacological, metabonomics, and/or clinical trial methods.

## Introduction

Liver disease, one of the most risk factors threatening human health, is the fifth most common cause of death worldwide after heart disease and stroke (Williams, 2006), mainly including alcoholic liver disease (ALD), non-alcoholic fatty liver disease (NAFLD), chronic viral hepatitis (e.g., hepatitis B virus and hepatitis C virus infections), autoimmune hepatitis (AIH), hepatic schistosomiasis (HS), liver cirrhosis (LC), hepatocellular carcinoma (HCC), and so forth (Wang et al., 2014). In recent years, several types of liver diseases received widespread attention and have become a public health problem. NAFLD, a reported prevalence of 6–35% worldwide (Federico et al., 2016), is often associated with the metabolic syndrome. At present, NAFLD has become an important cause of chronic liver disease in developed countries, and its incidence has been increasing significantly in recent years. HCC accounts for ~75% of liver cancer cases (Petrick et al., 2016). It is one of the most common malignant tumors in the world, especially in Asia, Africa, and Europe. According to World Health Organization (WHO) statistics, the mortality rate of HCC was as high as 95% in 2012. Moreover, hepatitis B virus (HBV) and hepatitis C virus (HCV) infections affect at least 2 and 150 million people worldwide, respectively (Wang et al., 2014).

In recent years, the incidence of various liver diseases among Tibetan people has been reported. Yan et al. (2007) found that the incidence of fatty liver was 29.89% among 696 cadres living in Tibet, China. The prevalence of ALD among adult Tibetans in Lhasa, Tibet, China is 4.87% (Baima et al., 2016). Zhao et al. (2001) reported a high prevalence of HBV infection in Tibet of China, and the average positive rate of hepatitis B surface antigen (HBsAg) (19.1%) among the native Tibetan population was much higher than that of China (10%) and the world (<5%). Moreover, liver cancer and gastric cancer are still the leading causes of cancer deaths in Tibet. These two kinds of cancer account for 75.2% of all cancer deaths in 2004–2005 (Li et al., 2011). On the other hand, hepatic echinococcosis, a common parasitic disease in pastoral areas, is mainly prevalent in Qinghai, Inner Mongolia, Sichuan, Xinjiang, and Tibet of China. The highest prevalence of echinococcosis in the world has been reported from the Tibetan plateau, China (Wang et al., 2008). In Tibet, the overall incidence of hepatic echinococcosis is 5–10% (Zhu et al., 2012). Schantz et al. (2003) reported that 6.6% of the Tibetan volunteers from Qinghai of China had confirmed infection with *Echinococcus granulosus*. The high incidence of some liver diseases among Tibetans may be related to their special dietary patterns. Due to the cold climate and hypoxia in the residential areas, they like to eat high-calorie foods (e.g., yak meat and mutton), while consuming less fruits and vegetables. Moreover, they love to drink barley wine and butter tea. Long-term high-fat and high-protein diet as well as drinking may contribute to the high incidence of fatty liver and ALD (Yan et al., 2007). Of course, other than dietary patterns, other risk factors need attention. For example, the high incidence of echinococcosis and HBV infection among Tibetan people can be attributed to their actual exposure to larval *Echinococcus* spp. and hepatitis B virus, respectively (Zhao et al., 2001; Zhu et al., 2012).

Traditional Tibetan medicine (TTM) is one of the world's oldest known medical systems. It has a long history of more than 2000 years. TTM originated from the local folk tradition called Bon that can be traced back to about 300 B.C. Later, TTM has gradually developed into a unique medical system by incorporating the theories of early traditional Chinese medicine, India medicine (Ayurveda), and Arabia medicine. The fundamental theory of TTM is three elements (also known as three humors) theory consisting of “*rLung*,” “*mKhris-pa*,” and “*Badkan*.” TTM believes that the three elements jointly maintain the body's physiological balance. Among them, *mKhris-pa* represents fire, helping digestion, accelerating the decomposition of waste, absorbing heat energy from food, and producing heat energy (Luo et al., 2015), and so is the source of many functions such as thermoregulation, metabolism, and liver function. In Qinghai-Tibet Plateau of China, TTM plays an important role in the health care system. It has been practiced by Tibetan physicians throughout the Tibetan regions, including Tibet, Qinghai, Gannan State of Gansu, Ganzi State and Aba State of Sichuan, and Diqing State of Yunnan. The number of physicians practicing TTM was over 5,000 (Luo et al., 2015). Similar to traditional Chinese medicine, TTM mainly uses herbs, animals and sometimes minerals to treat diseases. According to the latest statistics (Jia and Zhang, 2016), 3,105 natural medicines including 2,644 plants, 321 animals, and 140 minerals have been used in Tibetan medicine system. TTM has long-term clinical practices and accumulated rich experience in the treatment of various diseases. It has proved particularly beneficial in the treatment of chronic diseases, such as hepatitis, high altitude polycythemia, gastritis, stroke, cholecystitis, and rheumatism. It is worth noting that TTM has been widely used for the treatment of liver diseases in clinical practice. Many TTM monographs and official drug standards recorded a lot of natural medicines and prescriptions that were traditionally used to treat a variety of liver diseases. However, most of these records are scattered, and lack of systematic summary and induction.

In this paper, a bibliographic investigation of TTM monographs and drug standards and data mining were performed to sample information on natural Tibetan medicines used to treat liver diseases. Their names, original species, families, medicinal parts, treated diseases, and reported biological activities are shown in detail. These data can provide a good reference for their development and utilization. Moreover, we reviewed the most frequently used TTM in terms of their original species, traditional uses, active ingredients, and biological/pharmacological activities. These natural medicines might be a valuable gift from the old Tibetan medicine to the world, and would be potential drug candidates for the treatment of liver diseases.

## Methods

We manually searched 22 Tibetan medicine monographs and drug standards (Table S1), such as “Jing Zhu Materia Medica,” “Dictionary of Chinese Ethnic Medicine,” “Drug Standards of Tibetan Medicine,” “Tibetan Medicine Annals,” “Annotation of Commonly Used Tibetan Medicine Prescription,” and “Chinese Herbalism for Tibetan Medicine,” to obtain the information on natural Tibetan medicines and their prescriptions used for the treatment of a variety of liver diseases. Data collected from these literatures included names, original species, families, medicinal parts, and treated diseases. The botanical names of original plants are mainly from the references, and verified through the “Flora of China (http://frps.eflora.cn/)” database based on their Chinese names. The database of “The Plant List (http://www.theplantlist.org/)” is also used to standardize their Latin names.

In order to know the most frequently used Tibetan medicines for the treatment of liver diseases, data mining was performed to obtain the usage frequency of each medicine in traditional Tibetan prescriptions by using Traditional Chinese Medicine Inheritance Support System (TCMISS) (Version 2.5) (Yan et al., 2016). All collected prescriptions were manually entered into the TCMISS software, and the usage frequency of each medicine was ranked from large to small by clicking on the “Frequency Statistics” module. In addition, we searched the online Chinese databases (e.g., Wanfang, Weipu, and CNKI) and international databases (e.g., ISI Web of Science, MEDLINE, Science Direct, and Google Scholar) to obtain the active ingredients and biological/pharmacological effects of the selected species using their vernacular, English, or Latin names as search keywords.

## Results and discussion

### Traditional tibetan medicines documentation for the treatment of liver diseases

This paper recorded the uses of 193 species of natural Tibetan medicines for the treatment of various liver diseases in the traditional Tibetan medical system. The scientific name, family, medicinal part, treated disease, and reported biological activities of these natural medicines are given in Table 1 and Table S2 (see Supplementary Material). These medicines were distributed among 54 families. The most common families are: Gentianaceae (14%), Compositae (12%), Papaveraceae (9%), Labiatae (6%), Saxifragaceae (6%), Ranunculaceae (5%), Scrophulariaceae (4%), and Leguminosae (4%) (Figure S1). Moreover, herb (71%) is the primary source of these medicinal plant species, followed by tree and shrub (9% each), animal (7%), vine and mineral (3% each), lichen and fungus (1% each) (Figure S2). Among various plant parts used, the whole plant was the most frequently used (39%), followed by root and flower (9% each), fruit and aerial part (8% each), seed (7%), rhizome and inflorescence (2% each) (Figure S3).

**Table 1:** The most frequently used Tibetan medicines for the treatment of liver diseases in traditional Tibetan medical system.

**No**.	**Latin name**	**Tibetan name**	**Family**	**Life form**	**Used part**	**Treated liver diseases**	**Reported biological activities associated with liver diseases**	**Used frequency**
1	*Carthamus tinctorius* L.	Ku-gong ( 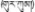 )	Compositae	Herb	Flower	Hepatitis, and hepatic heat (Editorial Board of Chinese Ethnic Medicine, 1990; Qinghai Institute for Drug Control, 1996; Dimaer, 2012)	Hepatoprotective, antioxidant, anti-inflammatory, suppressing the development of liver cell carcinoma, and anti-hepatic fibrosis activities (Okuno et al., 1998; Jun et al., 2011; Wu S. et al., 2013; Zhou et al., 2014; Hu and Wang, 2015)	125 (41.25%)
2	A natural exudate oozed from rock stratum, sometimes containing animal feces	Brag-zhun ( 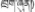 )	–	–	–	Chronic liver diseases, hepatic heat and hepatitis (Dimaer, 2012; Jia and Zhang, 2016)	Hepatoprotective, antioxidant and anticancer effects (Pant et al., 2016; Wang et al., 2016; Ye et al., 2017a,b)	76 (25.08%)
3	*Swertia chirayita* (Roxb. ex Flem.) Karst.	Di-da ( 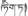 )	Gentianaceae	Herb	Whole plant	Icterohepatitis and viral hepatitis (Chinese Pharmacopoeia Commission, 1995; Jia and Zhang, 2016)	Antioxidant, hepatoprotective, anti-inflammatory, and anti-hepatitis B virus activities (Brahmachari et al., 2004; Joshi and Dhawan, 2005; Kumar and Van Staden, 2015)	75 (24.75%)
4	*Swertia mussotii* Franch	Di-da ( 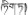 )	Gentianaceae	Herb	Whole plant	Icterohepatitis and viral hepatitis (Editorial Board of Chinese Ethnic Medicine, 1984; Chinese Pharmacopoeia Commission, 1995; Luo, 1997)	Hepatoprotective and anti-hepatitis B virus effects (Lv et al., 2010; Cao et al., 2013; Xu, 2013)	
5	*Swertia franchetiana* Harry Sm.	Di-da ( 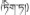 )	Gentianaceae	Herb	Whole plant	Liver diseases, icterohepatitis and viral hepatitis (Jia and Zhang, 2016)	Protective effect against liver injury caused by CCl_4_ (Zhang X. M. et al., 2011)	
6	*Halenia elliptica* D.Don	Di-da ( 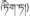 )	Gentianaceae	Herb	Aerial part	Icterohepatitis, hepatitis B and hepatitis (Editorial Board of Chinese Ethnic Medicine, 1984; Chinese Pharmacopoeia Commission, 1995; Dimaer, 2012)	Hepatoprotective, antioxidant, free radical-scavenging, anti-fatty liver, and anti-hepatitis B virus activities (Huang et al., 2010; Gu X. Y. et al., 2015; Zhang et al., 2015)	
7	*Herpetospermum pedunculosum* (Ser.) C.B. Clarke	Se-ji-mei-duo ( 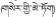 )	Cucurbitaceae	Herb	Seed	Icterohepatitis, liver heat and viral hepatitis (Yang, 1991; Chinese Pharmacopoeia Commission, 1995; Dimaer, 2012; Jia and Zhang, 2016)	Hepatoprotective, anti-hepatitis B virus, anti-inflammatory, and free radical scavenging effects (Yuan et al., 2006; Fang et al., 2007; Li et al., 2014; Liu and Zhang, 2016)	74 (24.42%)
8	*Phyllanthus emblica* L.	Ju-ru-re ( 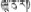 )	Euphorbiaceae	Tree	Fruit	Liver diseases (Editorial Board of Chinese Herbalism, 2002)	Hepatoprotective, antioxidant, anti-inflammatory, anti-hepatic fibrosis, anti-hepatitis B virus effects (Khan, 2009; Thilakchand et al., 2013); Anticancer activity against hepatocellular carcinoma cell (HepG2) (Ngamkitidechakul et al., 2010); Protective effect on high fat diet-induced non-alcoholic fatty liver disease (Huang et al., 2017)	71 (23.43%)
9	*Adhatoda vasica* Nees	Ba-xia-ga ( 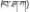 )	Acanthaceae	Shrub	Whole plant	Liver heat (Jia and Zhang, 2016)	Hepatoprotective effect (Bhattacharyya et al., 2005)	71 (23.43)
10	*Veronica ciliata* Fisch.	Ba-xia-ga ( 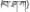 )	Scrophulariaceae	Shrub	Whole plant	Hepatitis (Luo, 1997; Jia and Zhang, 2016)	Antioxidant and hepatoprotective activities (Yin et al., 2014)	
11	*Bos taurus domesticus* Gmelin	Ge-wang ( 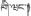 )	Bovidae	Animal	Gallstone	Hepatitis and hepatic echinococcosis (Luo, 1997; Dimaer, 2012)	Protective effect on ANIT-induced intrahepatic cholestasiss (Wu T. et al., 2013)	69 (22.77%)
12	*Meconopsis integrifolia* (Maxim.) Franch.	Wu-bai-en-bu ( 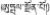 )	Papaveraceae	Herb	Whole plant	Hepatitis and liver heat (Qinghai Institute of Plateau Biology, 1975; Editorial Board of Chinese Herbalism, 2002; Dimaer, 2012)	Hepatoprotective and antioxidant activities (Zhou et al., 2013)	68 (22.44%)
13	*Meconopsis quintuplinervia* Regel	Wu-bai-en-bu ( 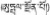 )	Papaveraceae	Herb	Whole plant	Hepatitis and liver heat (Chinese Pharmacopoeia Commission, 1995; Tian, 1997; Dimaer, 2012)	Hepatoprotective, anti-hepatic fibrosis, and antioxidant activities (He et al., 2012; Wang et al., 2013a,b)	
14	*Lagotis brevituba* Maxim.	Hong-lian ( 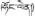 )	Scrophulariaceae	Herb	Whole plant	Acute and chronic hepatitis (Editorial Board of Chinese Ethnic Medicine, 1984; Jia and Zhang, 2016)	Protective effect on CCl_4_-induced acute hepatic injury (Zhu et al., 2016)	53 (17.49%)
15	*Lagotis integra* W.W. Sm.	Hong-lian ( 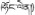 )	Scrophulariaceae	Herb	Whole plant	Acute and chronic hepatitis (Yunnan Editorial Board of Local Chronicles, 1995)	No report	
16	*Dracoephalum tanguticum* Maxim	Zhi-yang-gu ( 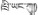 )	Labiatae	Herb	Aerial part or whole plant	Liver heat, hepatitis, and hepatomegaly (Chinese Pharmacopoeia Commission, 1995; Tian, 1997; Yunnan University of TCM, 2008; Dimaer, 2012)	Anti-hepatocarcinoma activity *in vitro* (Zheng et al., 2017), and protective effect on hypoxia-induced liver injury (Li et al., 2016)	48 (15.84%)
17	*Terminalia bellirica* (Gaertn.) Roxb.	Pa-ru-la ( 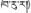 )	Combretaceae	Tree	Fruit	Liver diseases (Yunnan Editorial Board of Local Chronicles, 1995)	Hepatoprotective, antioxidant, and anti-hepatitis B virus activities (Qin, 2004; Lee et al., 2005; Tasduq et al., 2006; Bao et al., 2016)	44 (14.52%)
18	*Moschus berezovskii* Flerov	La-zai ( 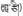 )	Cervidae	Animal	Dry secretions in the sachet	Hepatitis (Editorial Board of Chinese Herbalism, 2002; Jia and Zhang, 2016)	No report	39 (12.87%)
19	*Moschus sifanicus* Przewalski	La-zai ( 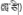 )	Cervidae	Animal	Dry secretions in the sachet	Hepatitis (Editorial Board of Chinese Herbalism, 2002; Jia and Zhang, 2016)	No report	
20	*Saxifraga umbellulata* var. *pectinata* (C. Marquand & Airy Shaw) J.T. Pan	Song-di ( 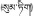 )	Saxifragaceae	Herb	Whole plant	Liver heat and hepatitis (Qinghai Institute for Drug Control, 1996)	Protective effect on ANIT-induced liver injury (Li et al., 2017)	34 (11.22%)
21	*Saxifraga umbellulata* Hook. f. & Thomson	Song-di ( 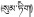 )	Saxifragaceae	Herb	Whole plant	Liver heat and hepatitis (Qinghai Institute of Plateau Biology, 1975; Qinghai Institute for Drug Control, 1996; Dimaer, 2012)	Protective effect on ANIT-induced liver injury (Li et al., 2017)	
22	*Crocus sativus* L.	Ge-er-geng ( 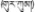 )	Iridaceae	Herb	Stigma	Liver diseases (Qinghai Institute for Drug Control, 1996; Editorial Board of Chinese Herbalism, 2002; Dimaer, 2012)	Hepatoprotective, anti-inflammatory, and anti-hepatic fibrosis activities (Hosseinzadeh and Younesi, 2002; Yang et al., 2009; Wang and Zhu, 2010)	27 (8.91%)
23	*Hypecoum leptocarpum* Hook. f. & Thomson	Ba-er-ba-da ( 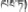 )	Papaveraceae	Herb	Whole plant	Hepatitis (Chinese Pharmacopoeia Commission, 1995; Luo, 1997)	Hepatoprotective effect (Zhang et al., 2016)	27 (8.91%)
24	*Hypecoum erectum* L.	Ba-er-ba-da ( 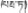 )	Papaveraceae	Herb	Whole plant	Hepatitis (Editorial Board of Chinese Ethnic Medicine, 1984; Tian, 1997)	Hepatoprotective effect (Nikolaev et al., 2014; Zhang et al., 2016)	
25	*Ursus thibetanus* G. Cuvier	Dong-chi ( 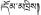 )	Ursidae	Animal	Gallbladder	Liver diseases (Yang and Chuchen, 1987; Editorial Board of Chinese Herbalism, 2002)	Anti-hepatic fibrosis, hepatoprotective, and anti-hepatocarcinoma activities (Zhou et al., 2012; Zhou C. F. et al., 2015)	27 (8.91%)
26	*Ursus arctos* L.	Dong-chi ( 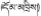 )	Ursidae	Animal	Gallbladder	Liver diseases (Ye and Guo, 1998; Jia and Zhang, 2016)		
27	*Tinospora sinensis* (Lour.) Merr.	Le-zhe ( 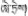 )	Menispermaceae	Vine	Rattan cane	Hepatitis and liver heat (Dimaer, 2012; Jia and Zhang, 2016)	Hepatoprotective effect (Nagarkar et al., 2013)	23 (7.59%)

By comparison with other articles, we found that most of the species used in the Tibetan medical system to cure liver diseases are different from those reported elsewhere. Chassagne et al. (2017) reported 83 species commonly used by Khmer traditional healers to treat liver disorders in Phnom Penh area, Cambodia. Only one species (i.e., *Oroxylum indicum*) has a similar use to our article. Similarly, Mukazayire et al. (2011) described 86 herbs used in Southern Rwanda for the treatment of liver diseases. Only one species mentioned by them is included in our study viz. *Bidens pilosa*. Moradi et al. (2016) reported 26 medicinal plants used for liver disorders in Iranian traditional medicine. Among them, only *Rheum palmatum* is also mentioned in the present article. In addition, 94, 99, 7 species were found for the treatment of liver diseases in the Southern Regions of Korea, the Maritime region of Togo and Nallamalais, Andhra Pradesh, India, respectively (Kim and Song, 2013; Sabjan et al., 2014; Kpodar et al., 2016). However, these species are quite different from the ones reported in our article. It may be due to the specific mountain flora. Most of the species (e.g., Brag-zhun, *Swertia mussotii, Halenia elliptica*, and *Herpetospermum pedunculosum*) reported in the present study are mainly distributed in Tibetan Plateau.

TTM has a unique understanding of the occurrence and development of liver disease. It believes that the pathogenesis of liver disease is divided into external and internal causes (Yutuo, 1983). The external causes include dampness, heat, and epidemic toxin (similar to western medicine's virus), while the internal causes refer to improper diet and overwork (Xizhu, 2010). Long-term or excessive consumption of alcoholic beverages, or salty, sour, spicy, moldy, and greasy foods, or strong work and strenuous exercise will cause the disorder of several basic substances including *rLung, mKhris-pa, Badkan*, and blood in the body (Bianba and Basang, 2000). Among them, the *mKhris-pa* disorder will lead to heat toxin invasion of the liver, and eventually cause a variety of liver diseases. In traditional Tibetan medical system, liver disease is divided into several types, such as jaundice liver disease, toxic hepatopathy, and liver heat (Xizhu, 2010). Among them, the liver heat belongs to the category of “*mKhris-pa*” disease, and its nature belongs to fire. Symptoms of liver heat disease mainly include loss of appetite, liver, and stomach discomfort, abdominal distension, dislike greasy food, and fatigue.

**Figure 1:**
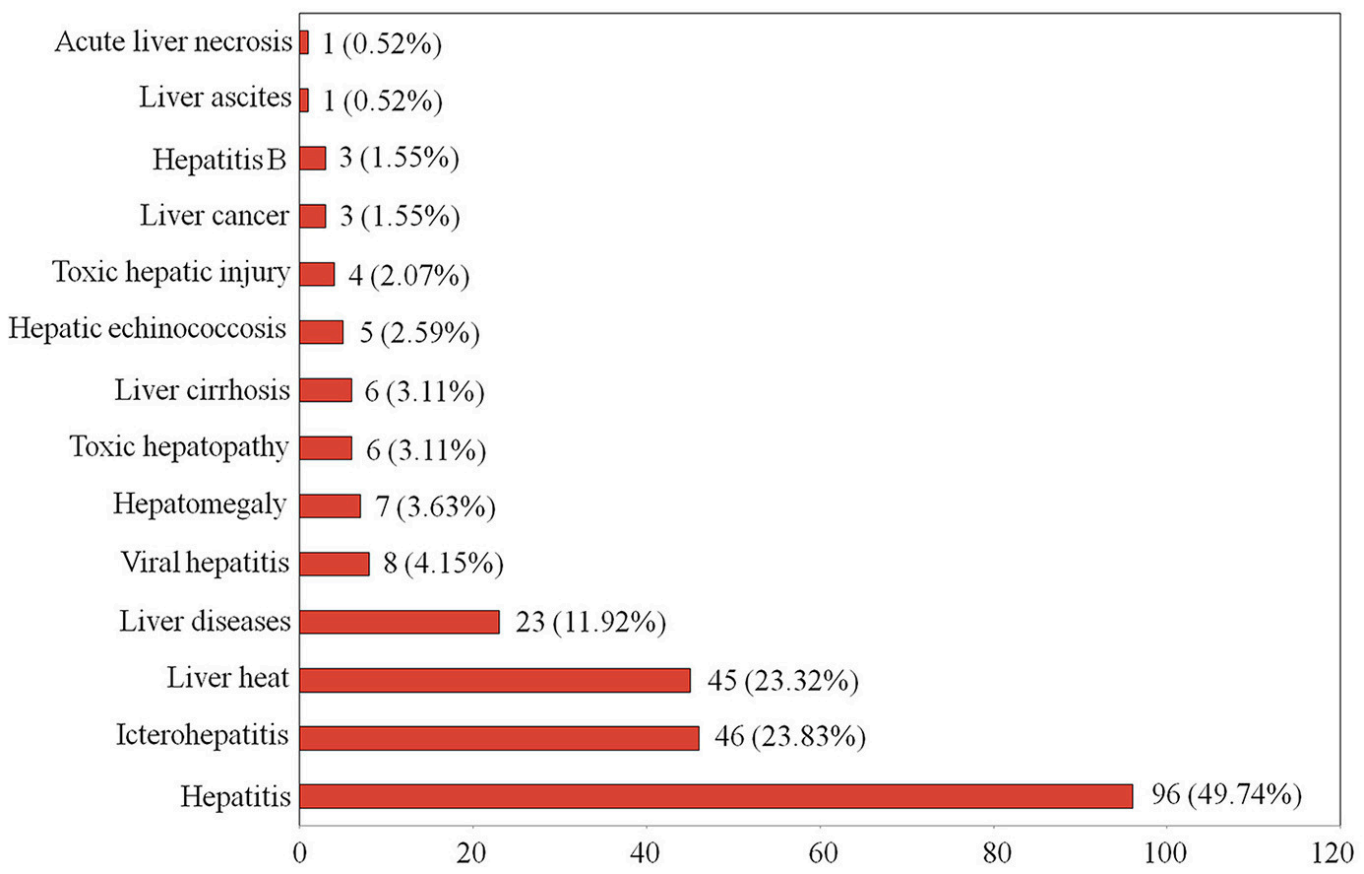
The number and percentage of traditional Tibetan medicines used in the treatment of various liver diseases.

In the present study, we found that 193 Tibetan medicines were used to treat various liver diseases, such as hepatitis, hepatomegaly, viral hepatitis, hepatitis B, toxic hepatopathy, liver heat, and so forth (Figure 1). The results indicated that some species were documented to treat broad liver diseases (e.g., hepatitis and liver heat), while some were clearly indicated for the treatment of specific liver diseases (e.g., hepatitis B). In order to ensure the primordial nature of the information obtained from 22 monographs, we did not incorporate similar disease types in statistics, although the scope of some liver diseases cross each other. For example, viral hepatitis includes hepatitis B, but we still do separate statistics on the two in Figure 1. For liver diseases therapy, the Tibetan people preferred natural medicines most frequently for the treatment of hepatitis using 96 species (37.65%), 46 for icterohepatitis (18.04%), and 45 for liver heat (17.65%) (Figure 1). There were 23 species (9.02%) recorded in the treatment of extensive liver diseases. In addition, 8, 7, and 6 species were described to be able to treat viral hepatitis, hepatomegaly and toxic hepatopathy, respectively. It is noteworthy that 5 *Chrysosplenium* plants (*Chrysosplenium carnosum, Chrysosplenium nudicaule, Chrysosplenium griffithii, Chrysosplenium lanuginosum*, and *Chrysosplenium nepalense*) and *Sphaerophysa salsula* were used to cure liver cirrhosis. More importantly, 3 *Rhododendron* plants (*Rhododendron anthopogon, Rhododendron anthopogonoides*, and *Rhododendron primuliflorum*) were clearly indicated for the treatment of liver cancer, and three species (*H. elliptica, Coriolus versicolor*, and *Halenia corniculata*) for hepatitis B. These information are of great value for the development of potential candidate drugs.

Out of 193 species traditionally used for liver diseases treatment, 51 species (26.42%) have been experimentally demonstrated to have various biological and pharmacological activities associated with liver diseases (Table 1 and Table S2), such as the hepatoprotective, anti-hepatic fibrosis, antioxidant, anti-inflammatory, and anti-hepatitis B virus effects. These findings have proved the validity of these species traditionally used in the treatment of liver diseases. So far, however, there are still 142 species (73.58%) that lack modern experimental evidences. Therefore, more in-depth studies are necessary in order to make better use of these traditional Tibetan medicines.

### The most frequently used natural medicines in traditional tibetan medical system for liver diseases treatment

**Figure 2:**
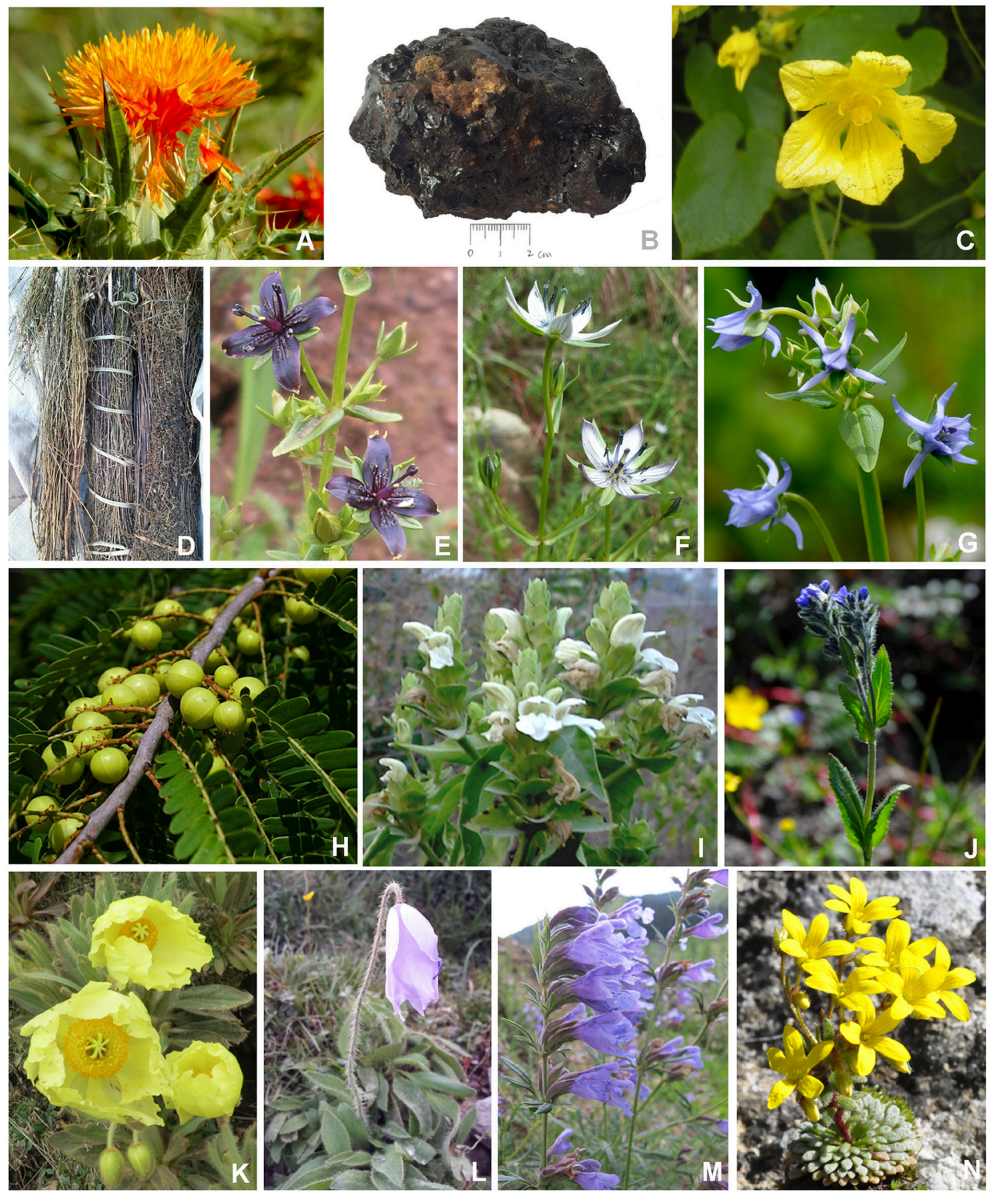
The most frequently used natural Tibetan medicines for the treatment of liver diseases in traditional Tibetan medical system. **(A)** *Carthamus tinctorius*, **(B)** Brag-zhun, **(C)** *Herpetospermum caudigerum*, **(D)** *Swertia chirayita*, **(E)** *Swertia mussotii*, **(F)** *Swertia franchetiana*, **(G)** *Halenia elliptica*, **(H)** *Phyllanthus emblica*, **(I)** *Adhatoda vasica*, **(J)** *Veronica ciliata*, **(K)** *Meconopsis integrifolia*, **(L)** *Meconopsis quintuplinervia*, **(M)** *Dracoephalum tanguticum*, and **(N)** *Saxifraga umbellulata* var. *pectinata*.

In order to know the most frequently used Tibetan medicines for the treatment of liver diseases, data mining based on TCMISS software was performed to obtain the usage frequency of these medicines in traditional Tibetan prescriptions. As a result, 303 prescriptions for the treatment of liver diseases were collected from Tibetan monographs and drug standards. Species with used frequencies above 20 are shown in Table 1 and Figure 2. The top five Tibetan medicines are Ku-gong (*Carthamus tinctorius*) with the used frequency of 125, Brag-zhun with 76, Di-da (*Swertia chirayita, S. mussotii, S. franchetiana*, and *H. elliptica*) with 75, Se-ji-mei-duo (*H. pedunculosum*) with 74, and Ju-ru-re (*Phyllanthus emblica*) with 71. In the following sections, the names, original species, traditional uses, active ingredients, and biological/pharmacological activities of these five Tibetan medicines have been summarized in detail.

### Carthamus tinctorius

The dried flower of *C. tinctorius*, known as Ku-gong (Tibetan: 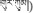), Honghua (Chinese name) or safflower (English name), is a commonly used herbal medicine. In China, *C. tinctorius* is widely cultivated in various locations of Henan, Sichuan, Xinjiang, and Zhejiang provinces. In the traditional theory of Tibetan medicine, Ku-gong is pungent in flavor and warm in nature (Health Bureau of Tibet et al., 1979). It is applied for the treatment of dysmenorrhea, dystocia, traumatic injury, blood stasis, hepatitis, and hepatic heat for thousands of years in Tibetan clinics (Health Bureau of Tibet et al., 1979). So far, many chemical constituents have been isolated from safflower, such as hydroxysafflor yellow A, safflor yellow A, luteolin, kaempferide, and adenosine (Zhou et al., 2014). Among them, hydroxysafflor yellow A is the major bioactive compound, and so is usually used as the marker for controlling the quality of safflower in pharmaceutical industry and drug standards.

Moreover, it is worth pointing out that phytochemicals or extracts obtained from the safflower have been proved to possess some pharmacological activities associated with liver diseases. For example, Zhang Y. et al. (2011) reported that hydroxysafflor yellow A showed an obvious protective effect against carbon tetrachloride-induced liver fibrosis in rats. Jiang et al. (2014) found that hydroxysafflor yellow A can reduce ischemia/reperfusion-induced acute liver injury by directly attenuating macrophage activation under inflammatory conditions. Carthamus red isolated from safflower was found to have strong antioxidant and hepatoprotective effects against CCl_4_-induced liver damage in rats (Wu S. et al., 2013). Moreover, the methanol extract of *C. tinctorius* was demonstrated to have anti-inflammatory action by inducing heme oxygenase-1 expression via NF-E2-related factor translocation and inhibiting NF-κB activity (Jun et al., 2011). Hu and Wang (2015) reported that the water extract of *C. tinctorius* has significant inhibitory effect on diethylnitrosamine-induced liver cirrhosis in rats. Besides, safflower oil, rich in n-6 polyunsaturated fatty acids, was reported to be able to alter the membrane fatty acid composition of the liver, and suppress the development of liver cell carcinoma induced by diethylnitrosamine in rats (Okuno et al., 1998). These results suggest that Ku-gong (safflower) may serve as a drug candidate for various liver diseases treatment.

### Brag-zhun

Brag-zhun (Tibetan: 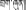), one of the commonly used Tibetan medicines, is a well-known natural medicine all over the world recorded by other names, such as Mineral pitch in English, *Asphaltum punjabianum* in Latin, also known as Baragshun (Mongolian), Tasmayi (Kazakh), Mumlai (Farsi), Shilajit (Sanskrit), Shilajeet (Hindi), and Moomiyo (Russian). It is found predominately in the Altai, Himalaya, and Caucasus mountains. In Ayurveda, the traditional Indian system of medicine, shilajit has been recognized as a rejuvenator because it can prevent ailment and enhance the quality of life (Wilson et al., 2011).

Brag-zhun was first recorded in the classic Tibetan book “Yue Wang Yao Zhen (Somaratsa)” compiled in the mid-eighth century. In China, brag-zhun is mainly distributed in the Qinghai-Tibet Plateau, such as Tibet, Qinghai, Ganzi, and Aba of Sichuan, and Shangri-La of Yunnan. There are several schools of thought for the origin of brag-zhun. Most researchers think that brag-zhun is a natural exudate oozed from rock stratum, sometimes containing animal feces (e.g., *Trogopterus xanthotis, Ochotona erythrotis*; Wilson et al., 2011; Cao et al., 2016). In China, brag-zhun as well as its preparations are commonly used Tibetan medicines for the treatment of hot diseases, especially good at treating liver (e.g., hepatitis, hepatomegaly) and ocular diseases (e.g., conjunctivitis) (Chinese Pharmacopoeia Commission, 1995; Cao et al., 2016).

It has been reported that brag-zhun contains a lot of minerals in ionic form, as well as organic matter (e.g., humic acid and fulvic acid). Moreover, carotenoids, indigoids, amino acids, essential fatty acids, and vitamins were also found in brag-zhun (Wilson et al., 2011; Cao et al., 2015). Modern pharmacological study has demonstrated that the n-butanol extract of brag-zhun has an obvious protective effect against acetaminophen-induced acute liver injury in mice (Ye et al., 2017a). Wang et al. found that the n-butanol extract of brag-zhun significantly reduced the serum levels of ALT, AST, TNF-α, and IFN-γ, increased the activity of SOD in liver tissue, decreased the activation levels of Caspase-3 and Caspase-8 in liver tissue, and reduced the pathological damage of liver tissue in mice with liver injury induced by concanavalin A. It indicated that the mechanisms of the hepatoprotective effect of brag-zhun may be related to the inhibition of release of inflammatory factors, antioxidant activity, and anti-apoptosis (Wang et al., 2016). In addition, brag-zhun as well as its water extract were reported to be able to significantly reduce the liver index and the serum levels of ALT and AST in mice with liver injury initiated by concanavalin A (Ye et al., 2017b). Recently, Pant et al. (2016) reported that mineral pitch (the same substance with brag-zhun) induced apoptosis via the production of ROS, and inhibited proliferation by modulating the expression levels of miRNA-21 and miRNA-22 in hepatic cancer cells (Huh-7).

### *Swertia chirayita, S. mussotii, S. franchetiana*, and *Halenia elliptica*

Di-da (Tibetan: 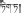) is a widely used traditional Tibetan medicine in China. It originates from multiple plant species. *S. chirayita, S. mussotii, S. franchetiana*, and *H. elliptica* are the most commonly used species of Di-da in China. In traditional theory, Di-da is bitter in flavor and cold in property (Health Bureau of Tibet et al., 1979). It can clear liver and gallbladder heat, and remove jaundice, and so has been commonly used for the treatment of liver and gallbladder diseases, such as icterohepatitis, viral hepatitis, and cholecystitis.

*Swertia chirayita*, a well-known herbal medicine in India, Nepal, and China, is widely grown in the temperate regions of Himalayas. Its medicinal usage is reported in various traditional systems of medicines, such as the Tibetan medicine, Ayurveda, Unani, and Siddha under different names (e.g., *Swertia chirata*, Chiretta, Anaryatikta, Chirrato, and Cherayata; Joshi and Dhawan, 2005). In China, *S. chirayita* is frequently used to treat damp-heat and quench the fire of the liver and gallbladder in traditional Tibetan medicine system (Fan et al., 2014). Modern investigations have shown that *S. chirayita* possesses a wide range of biological activities, such as hepatoprotective, anticancer, anti-inflammatory, hypoglycemic, antibacterial, and antiviral activities (Brahmachari et al., 2004; Joshi and Dhawan, 2005; Kumar and Van Staden, 2015). It is noteworthy that the extract of *S. chirayita* was reported to show protection against hepatotoxicity induced by paracetamol (Nagalekshmi et al., 2011). Mukherjee et al. (1997) found that different doses of *S. chirata* could improve the liver injury induced by CCl_4_ in albino rats, and the moderate dose (50 mg/kg body wt) was found to be the most effective. Moreover, the 50% EtOH–H_2_O extract of *S. chirayita* was reported to be able to inhibit the secretions of HBsAg and hepatitis B e antigen (HBeAg) (Zhou N. et al., 2015). These pharmacological activities are mainly attributed to the presence of a diverse group of bioactive phytochemicals in *S. chirayita*, such as swertiamarin, sweroside, gentiopicrin, mangiferin, amaroswerin, oleanolic acid, 3,3′,5-trihydroxybiphenyl, (+)-cycloolivil-4′-O-β-D-glucopyranoside, 1-hydroxy-3,7,8-trimethoxy xanthone, and 1,5,8-trihydroxy-3-methoxy xanthone. Among them, swertiamarin was reported to possess significant antioxidant and hepatoprotective effects against D-galactosamine induced acute liver damage (Jaishree and Badami, 2010). Gentiopicrin could decrease the serum ALT and AST levels, and increase the liver GSH-Px activity in the mice treated with CCl_4_, indicating significant liver protection property (Liu et al., 2002). Mangiferin showed significant hepatoprotective activity against D-galactosamine induced hepatotoxicity in rats via Nrf2–NFκB pathways (Das et al., 2012). In addition, (+)-cycloolivil-4′-O-β-D-glucopyranoside showed inhibitory activity on HBsAg secretion with IC_50_ values of 0.31 ± 0.045 mM (Zhou N. et al., 2015).

*Swertia mussotii* is an important and frequently used Tibetan medicinal plant indigenous to the Qinghai-Tibet Plateau. It is mainly distributed in high altitude areas (2000–3800 m) of Sichuan, Qinghai, Tibet, and Yunnan provinces in China. Qinghai's Yushu and northwest Sichuan plateau are the main producing areas of *S. mussotii*. In “Drug Standards of Tibetan Medicine,” *S. mussotii* was recorded with detoxification and clearing liver and gallbladder heat effects. It has been commonly used for the treatment of hepatitis, acute icterohepatitis, cholecystitis, epidemic fever, headache, and blood diseases in traditional Tibetan medicine system. Modern pharmacological experiment has proved that *S. mussotii* could alleviate the damage of immunological liver injury in mice caused by BCG vaccine and lipopolysaccharide (Xu, 2013). Lv et al. (2010) found that 75% ethanol extract of *S. mussotii* showed hepatoprotective effect on CCl_4_-induced acute liver damage in mice. Moreover, the alcohol extract of *S. mussotii* was reported to significantly reduce lipopolysaccharide-induced cholestatic liver damage in rats (Gao et al., 2014). Similar to *S. chirayita*, iridoid glycosides, xanthones, and triterpenoids are the major bioactive compounds in *S. mussotii*. The total iridoids and xanthones extracted from *S. mussotii* exhibited significant hepatoprotective effect on alpha naphthylisot hiocyanate-induced liver damage in mice (Tian et al., 2014). In addition, Cao et al. (2013) isolated several xanthones from *S. mussotii*, which exhibited significant anti-hepatitis B virus activity.

*Swertia franchetiana*, an annual herb, is also widely grown in the Qinghai-Tibet Plateau of China (Tibet, Sichuan, Qinghai, and southern Gansu) at elevations of 2200–3600 m. The whole plant of *S. franchetiana* is commonly used by Tibetan people for the treatment of various liver diseases, mainly icterohepatitis and viral hepatitis (Jia and Zhang, 2016). In recent years, multiple active phytochemicals belonging to different classes were isolated from *S. franchetiana*, such as swertiamarin, gentiopicrin, mangiferin, swertianolin, and oleanolic acid. It is worth pointing out that the content of swertiamarin was found to be higher in *S. franchetiana* than that in *S. chirata* and *S. mussotii* (Fan, 2012). Up to now, the pharmacological activities of *S. franchetiana* have rarely been reported. Zhang X. M. et al. (2011) found that the *n*-butanol extract of *S. Franchetiana* could exert a protective effect against liver injury caused by CCl_4_.

*Halenia elliptica* is a commonly used traditional herbal medicine. It is mainly distributed across the Tibet, Yunnan, Sichuan, Qinghai, Xinjiang, and Inner Mongolia of China as well as Nepal, Bhutan, and India. In “Drug Standards of Tibetan Medicine,” *H. elliptica* was described with clearing heat, promoting diuresis, calming the liver, and promoting bile flow functions. Its aerial part was used for the treatment of acute icterohepatitis, cholecystitis, dizziness, headache, and toothache in Tibetan clinics. The widespread uses of *H. elliptica* in traditional Tibetan medicine have resulted in considerable pharmacological and phytochemical studies of the plant. Huang et al. (2010) reported that administration of the methanolic extract of *H. elliptica* significantly decreased the serum levels of ALT, AST, ALP, and total bilirubin in rats with liver toxicity caused by CCl_4_. Similarly, the ethanol extract of *H. elliptica* was found to have hepatoprotective effect on CCl_4_-induced chemical hepatic injury in mice (Jin et al., 2007). The main active constituents of *H. elliptica* are recognized as xanthones (e.g., 1-hydroxy-2,3,5-trimethoxy xanthone and 1-hydroxy-2,3,4,5-tetramethoxy xanthone), flavonoids (e.g., luteolin and apigenin), and pentacyclic triterpenes (e.g., oleanolic acid) (Zhang et al., 2015). Gu X. Y. et al. (2015) reported that the total flavonoids isolated from *H. elliptica* could improve liver cell damage, decrease the serum level of MDA, and increase SOD level in rats with experimental steatohepatitis induced by high-fat diet.

### Herpetospermum pedunculosum

The dried seeds of *H. pedunculosum*, known as “Se-ji-mei-duo” (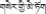) in Tibetan or “Bolengguazi” in Chinese, is one of the most representative Tibetan medicines. It is bitter in flavor and cold in property, and frequently used in the traditional Tibetan system of medicine for the treatment of icterohepatitis, liver heat, viral hepatitis, cholecystitis, and dyspepsia in the past few decades. *H. pedunculosum*, also named *H. caudigerum* in some literatures, belongs to the Cucurbitaceae family and is widely distributed in Tibet and Yunnan of China, India and Nepal at elevations of about 2300–3500 m.

Previous phytochemical studies have shown that the seeds of *H. pedunculosum* is rich in lignan compounds (e.g., herpetrione, herpetin, herpetetrone, herpetone, herpetradione, herpepropenal, herpetal, and herpepentol; Liu and Zhang, 2016). They have been proved to possess multiple pharmacological activities such as anti-hepatitis B virus, anti-inflammatory and hepatoprotective effects (Yuan et al., 2006; Yu et al., 2014; Shen et al., 2015; Liu and Zhang, 2016). It was reported that herpetrione displayed promising inhibitory potential against hepatitis b virus, which could reduce the replication and expression of HBsAg and HBeAg (Yuan et al., 2006). Moreover, herpetrione was discovered to be the active ingredient in protecting liver and lowering aminotransferase levels, and its nanosuspensions exhibited a significant hepatoprotective effect against acute liver injury induced by CCl_4_ in mice (Shen et al., 2013). Similarly, herpetin was found to have significant inhibitory effect on HBV-DNA *in vitro* (Yuan et al., 2006). Recently, Gu J. et al. (2015) reported that herpetin exhibited certain hepatoprotective activity against carbon tetrachloride-induced liver injury in mice, and this effect could be promoted through pharmaceutical application of liposome.

Moreover, in order to make better use of the seeds of *H. pedunculosum* in the treatment of liver diseases, some modern pharmacological studies were performed. Li et al. (2014) reported that administration of the seed oil of *H. pedunculosum* could significantly reduce CCl_4_-induced liver damage, decrease the serum levels of triglycerides, malondialdehyde, total bilirubin, and hepatic enzyme markers (e.g., alanine aminotransferase, aspartate aminotransferase, and alkaline phosphatase), and increase the activities of SOD in rats. The ethyl acetate extract of *H. caudigerum* was found to show a protective effect on CCl_4_-induced acute liver injury in mice (Shen et al., 2015). Besides, the total lignans extracted from *H. pedunculosun* seeds were found to have hepatoprotective effect on concanavalin A-induced immunological liver injury in mice (Gu et al., 2014). These findings mentioned above support the traditional applications of *H. pedunculosun* seeds by Tibetan medicine practitioners in liver diseases treatment.

### Phyllanthus emblica

The dried fruits of *P. emblica*, known as “Ju-ru-re” (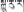) in Tibetan, is one of the commonly used Tibetan medicines in China. It is a well-known traditional medicine all over the world recorded by several names, such as *Emblica officinalis*, Indian gooseberry, Emblic myrobalan, Amla (Hindi), Amba (Nepalese), and Mirabolano emblico (Portuguese) (Khan, 2009). In Indian indigenous system of medicine (e.g., Ayurveda), Amla, also named as *E. officinalis* or Indian gooseberry, enjoys an important position because of its good resistance to a variety of diseases. Most of the parts of *E. officinalis* are used for medicinal purposes, for example, the fruits have been widely used in Ayurveda for the treatment of liver disorders, diabetes, diarrhea, jaundice, and inflammation (Khan, 2009; Krishnaveni and Mirunalini, 2010; Khosla and Sharma, 2012).

In traditional Tibetan system of medicine, Ju-ru-re is described as sweet, sour, astringent in flavor and cool in property. It is frequently used for the treatment of liver diseases, blood heat, sore throat, dry mouth, indigestion, abdominal pain, and cough for thousands of years (Health Bureau of Tibet et al., 1979; Chinese Pharmacopoeia Commission, 2015). *P. emblica* is a deciduous tree and widely grown and sometimes cultivated in subtropical and tropical areas including China, India, Sri Lanka, Indonesia, Malaysia, and Philippines. In China, *P. emblica* is mainly distributed in Sichuan, Guizhou, Fujian, Guangdong, Hainan, Guangxi, and Yunnan at elevations of 200–2300 m.

Up to now, many chemical constituents belonging to different classes have been isolated from the fruits of *P. emblica* including tannins, flavanoids, vitamins, amino acids, and carbohydrates. Hydrolyzable tannins (e.g., gallic acid, ellagic acid, corilagin, chebulagic acid, and geraniin) are the dominating active ingredients of the fruits of *P. emblica* (Yang and Liu, 2014). It was reported that gallic acid could protect the liver from injuries induced by various hepatotoxic agents, including paracetamol, sodium fluoride, cyclophosphamide, nitrosodiethylamine, and carbon tetrachloride in experimental animal models (Jadon et al., 2007; Rasool et al., 2010; Nabavi et al., 2013; Latief et al., 2016; Oyagbemi et al., 2016). Moreover, Hsu and Yen (2007) found that intake of gallic acid could be beneficial for the suppression of high fat diet-induced dyslipidaemia and hepatosteatosis in rats. Recently, gallic acid was found to ameliorate impaired glucose and lipid homeostasis in high fat diet-induced NAFLD mice by using ^1^H NMR-based metabolomics method (Chao et al., 2014). Similar to gallic acid, ellagic acid has also been reported to show obvious hepatoprotective effects in murine models against a variety of agents, such as paracetamol, carbon tetrachloride, alcohol, D-galactosamine, and concanavalin A (García-Nino and Zazueta, 2015). In addition, ellagic acid exhibited good antiviral properties against HBV and HCV (García-Nino and Zazueta, 2015). As another hydrolyzed tannin, corilagin was found to have hepatoprotective effect on galactosamine/lipopolysaccharide-induced liver injury in rats through suppression of oxidative stress and apoptosis (Kinoshita et al., 2007). Moreover, Hau et al. (2010) found that corilagin was considerably effective to retard the *in vivo* growth of xenografted Hep3B hepatocellular carcinoma. Given these beneficial biological activities, gallic acid, ellagic acid, and/or corilagin are usually qualitatively analyzed to evaluate the quality of Ju-ru-re by using HPLC method (Zhang et al., 2012). Moreover, the fruits of *P. emblica* are also rich in flavonoids, such as quercetin, kaempferol, rutin, quercetin 3-β-d-glucopyranoside, and kaempferol 3-β-d-glucopyranoside (Liu et al., 2008).

The widespread uses of the fruits of *P. emblica* in traditional medicines and food products have resulted in considerable pharmacological studies. A wide range of biological activities and potential health benefits have been reported, including hepatoprotective, gastroprotective, anti-inflammatory, anticancer, cardioprotective, and immunomodulatory effects (Khan, 2009; Krishnaveni and Mirunalini, 2010; Khosla and Sharma, 2012; Thilakchand et al., 2013; Yang and Liu, 2014). In this paper, we mainly focus on its hepatoprotective activity. *P. emblica* has been proven to have potential in protecting the liver from injuries induced by multiple hepatotoxic agents, such as ethanol, carbon tetrachloride, arsenic, ochratoxins, and antitubercular drugs in experimental models (Jose and Kuttan, 2000; Tasduq et al., 2005; Pramyothin et al., 2006; Panchabhai et al., 2008; Maiti et al., 2014). For exploring anti-viral compounds from *P. emblica*, eight new sesquiterpenoid glycosides along with three known compounds were isolated and evaluated for their anti-HBV activities. The results found that phyllaemblicin G6 displayed anti-HBV activities with IC_50_ of 8.53 ± 0.97 and 5.68 ± 1.75 μM for the HBsAg and HBeAg secretion, respectively (Lv et al., 2014). Recently, the water extract of *P. emblica* fruits was also found to show a protective effect on high fat diet-induced non-alcoholic fatty liver disease (NAFLD) in SD rats (Huang et al., 2017). In addition, pretreatment with the defatted methanolic extract of *E. officinalis* (100 and 200 mg/kg) significantly inhibited the appearance of γ-GT foci, pathological manifestations and tumor formation induced by the Solt-Farber regimen in the liver of Wistar rats (Sultana et al., 2008). The water extract of *P. emblica* fruits significantly decreased fat accumulation and ROS production in HepG2 cells, and also inhibited hepatic fibrosis in HSC-T6 cells (Lu et al., 2016). Besides, Ngamkitidechakul et al. (2010) found that the aqueous extract of *P. emblica* at 50–100 μg/mL significantly inhibited cell growth of six human cancer cell lines including hepatocellular carcinoma cell (HepG2). In summary, these modern pharmacological studies indicated that Ju-ru-re may have a good potential to develop into a candidate drug for the treatment of liver diseases.

### The potential toxicity of some traditional tibetan medicines

Although some positive effects of TTM in the treatment of liver diseases have been reported, attention should also be paid to the potential toxicity of some Tibetan medicines. For example, the aqueous extract of *C. tinctorius* was found to have toxic effects on mouse spermatogenesis (Mirhoseini et al., 2012). Nobakht et al. (2000) reported that the *C. tinctorius* extract displayed teratogenic effects on the central nervous system development in mice at the dose of 1.2 mg/kg/day, and its cytotoxic effect on the rat nervous cell culture appeared dose-dependent (Zhou et al., 2014). Besides, *C. tinctorius* is thought to be potentially harmful to pregnant women (Ernst, 2002).

Furthermore, we should also pay special attention to the hepatotoxicity of some Tibetan medicines. In our survey, Compositae was found as the second most commonly used family for liver diseases treatment, but some medicinal plants in this family (e.g., *Senecio scandens*) are known to contain pyrrolizidine alkaloids which can induce liver damage (Wang D. et al., 2013). It was reported that and the aqueous extract of *S. scandens* produced mild hepatotoxicity in rats at a high dose of 20 g/kg, and the toxicity was related to its total alkaloid content (Wang, 2008). Similarly, Lin et al. (2009) found that a single overdose (6 g/kg) of the water extract of *S. scandens* produced typical pyrrolizidine alkaloids-induced hepatotoxicity in rats. However, no significant hepatotoxic effects were observed in rats at the dose recommended by the Pharmacopeia of China. These findings indicated that the hepatotoxicity of *S. scandens* is dose related. Moreover, *Lithospermum erythrorhizon* was also reported to contain some pyrrolizidine alkaloids (Roeder and Rengel, 1990). Therefore, caution should be taken when administering *L. erythrorhizon* especially at high doses, although no significant toxicity has been observed so far in this herb (Han et al., 2015). In recent years, the safety of *Bupleurum chinense* and its products has raised general concerns. Lv et al. (2009) found that the long-term administration of the crude extract of *B. chinense* induced obvious hepatotoxicity in rats. It can not only cause the changes of liver function (ALT and AST), but also the pathological changes in hepatic cell. Besides, it is worth noting that *R. palmatum* was found to have bidirectional effects of liver protection and hepatotoxicity on CCl_4_-treated and normal rats. Its hepatotoxic effect could be attributable to the liver cell fibrosis induced by high doses of this herb (Wang et al., 2011).

In summary, despite the good benefits of Tibetan medicines in the treatment of liver diseases were indicated in our article, their potential toxicity should also be given enough attention. It is especially important to take precautions against drug-induced liver injury when selecting Tibetan medicines and their doses. More experiments should be encouraged to identify their side effects or toxicity so that they can be used safely and effectively.

## Concluding remarks

Nature medicines including medicinal plants, animals and minerals are nature's gift to human beings, which play an important role in the fight against various diseases. Many commonly used drugs of modern medicine have originated directly or indirectly from them, such as artemisinin, paclitaxel, and aspirin. Tibetan medicine is an important part of the world's traditional medical system. The Tibetan people living in the Qinghai-Tibet Plateau have accumulated rich experience in medication in their struggle against natural conditions and diseases. It is recognized that traditional Tibetan medicine has a good curative effect in the treatment of liver diseases, rheumatism, acute and chronic mountain sicknesses, cardiovascular and cerebrovascular diseases, and stomach diseases.

In the present study, we have attempted to generalize and congregate the names, original species, families, medicinal parts, traditional uses, and pharmacological information on natural Tibetan medicines traditionally used in the Tibetan system of medicine for liver diseases treatment. The results showed that these Tibetan medicines were mainly distributed among 54 families, and the most frequently used family is Gentianaceae. In addition, herb is the primary source of these medicines, and the whole plant is the most commonly used part. More importantly, we found several natural Tibetan medicines, such as *C. tinctorius*, Brag-zhun, *S. chirayita, S. mussotii, H. elliptica, H. pedunculosum*, and *P. emblica*, which were frequently used to treat liver diseases by bibliographic investigation and data mining. It is worth noting that *C. tinctorius* is also a widely used traditional Chinese medicine (TCM), which is mainly applied for blood-stasis syndrome with dysmenorrhea, amenorrhea, and postpartum abdominal pain in the clinical practice of TCM (Zhou et al., 2014). However, the present study found that *C. tinctorius* was frequently used to treat liver diseases in TTM system. The medicinal value of *C. tinctorius* in treating liver diseases deserves further exploration and utilization. Moreover, it is also need to pay special attention to several active compounds isolated from the most frequently used Tibetan herbs. We think that hydroxysafflor yellow A, swertiamarin, gentiopicrin, herpetrione, herpetin, gallic acid, and ellagic acid may be good and promising drug candidates for treating liver diseases because of their exact hepatoprotective effect as well as high concentration levels in the corresponding species. In order to assess the potential of these compounds for druggability, multidisciplinary approaches should be integrated to perform more pharmacological studies, reveal their mechanisms of action, clarify the pathways of their absorption, distribution, metabolism, and excretion (i.e., the ADME process), and also to assess their potential toxicity.

In addition, the gaps and limitations of current research on these Tibetan medicines also need to be pointed out. First, only 51 species (26.42%) have been demonstrated to have biological activities associated with liver diseases, and most species still lack adequately powered experimental evidences. For example, *Lagotis integra*, known as “Hong-lian” in Tibetan, is a commonly used Tibetan medicines for the treatment of liver diseases with the used frequency of 53. However, so far, no biological activities or active components related to liver diseases have been reported for this herb. Similarly, *Moschus berezovskii* and *M. sifanicus*, named “La-zai” in Tibetan with the used frequency of 39, also lack the liver disease-related study. Given their high frequency of use, these gaps of research need to be tackled urgently. Secondly, although some compounds isolated from Tibetan medicines were found to have biological activities associated with liver diseases, their mechanisms of action and possible synergies with each other have not been explicit enough. Further research is necessary to solve these problems. On the other hand, although some Tibetan herbal medicines (e.g., *P. emblica*) have been reported to inhibit the proliferation of several human liver cancer cell lines, these *in vitro* studies are definitely not sufficient to show that they have a positive effect on the treatment of hepatocellular carcinoma because the response of a cell line to an herbal extract may or may not occur in humans (Gertsch, 2009). Rigorous *in vivo* experiments and even clinical studies involving different mechanisms are still needed to confirm their effectiveness in the treatment of liver diseases.

In conclusion, this study provides the first compilation of data for the ethnomedicinal knowledge of TTM in the treatment of liver diseases. The medicinal species with high frequency of use may signpost the probable existence of valuable active compounds. In order to better develop and utilize these traditional Tibetan medicines, more efforts should be made to evaluate their biological activities *in vivo*, identify bioactive components, elucidate the underlying mechanism of action, and clarify their side effects or toxicity by using pharmacological, phytochemical, metabonomics, and/or clinical trial methods.

## Author contributions

QL: conducted the research, performed data analysis, and wrote the paper; H-JL, HD, TX: collected, organized, and analyzed the data; C-LH: wrote the Tibetan names of natural medicines; GF: conceived and designed the study; and YZ: amended the paper.

### Conflict of interest statement

The authors declare that the research was conducted in the absence of any commercial or financial relationships that could be construed as a potential conflict of interest.
