# Supplementary material for: Natural Medicines Used in the Traditional Tibetan Medical System for the Treatment of Liver Diseases
Source: Front Pharmacol. 2018 Jan 30;9:29. doi: 10.3389/fphar.2018.00029 (PMC5797630; doi:10.3389/fphar.2018.00029)
Supplement: Supplementary file 1 [file DataSheet1.doc]

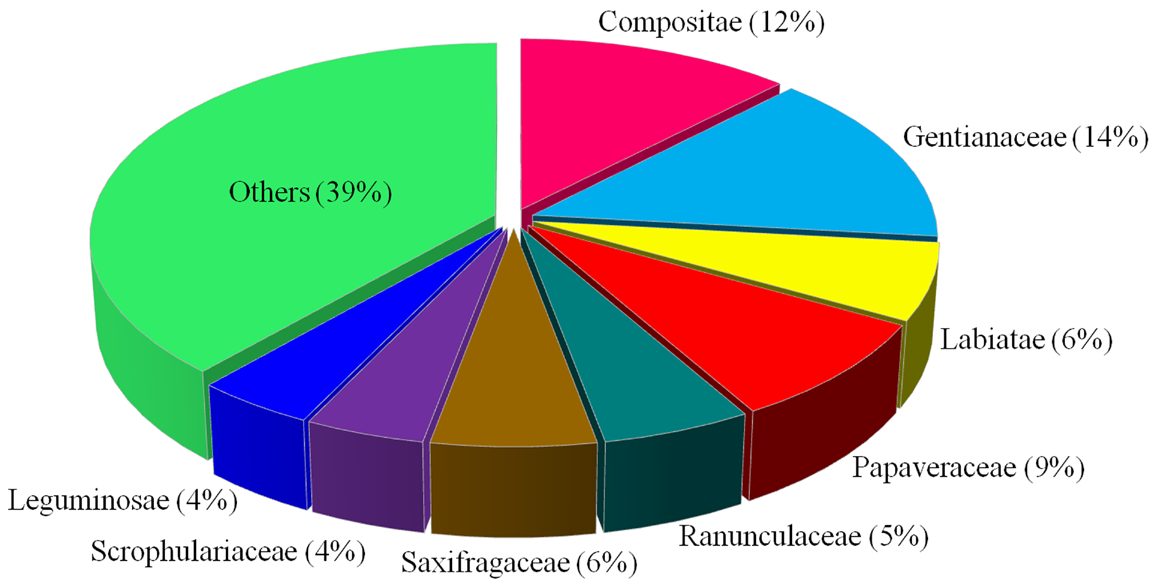


Supplementary Figure S1 Families distribution of traditional Tibetan medicines used in the treatment of liver diseases.


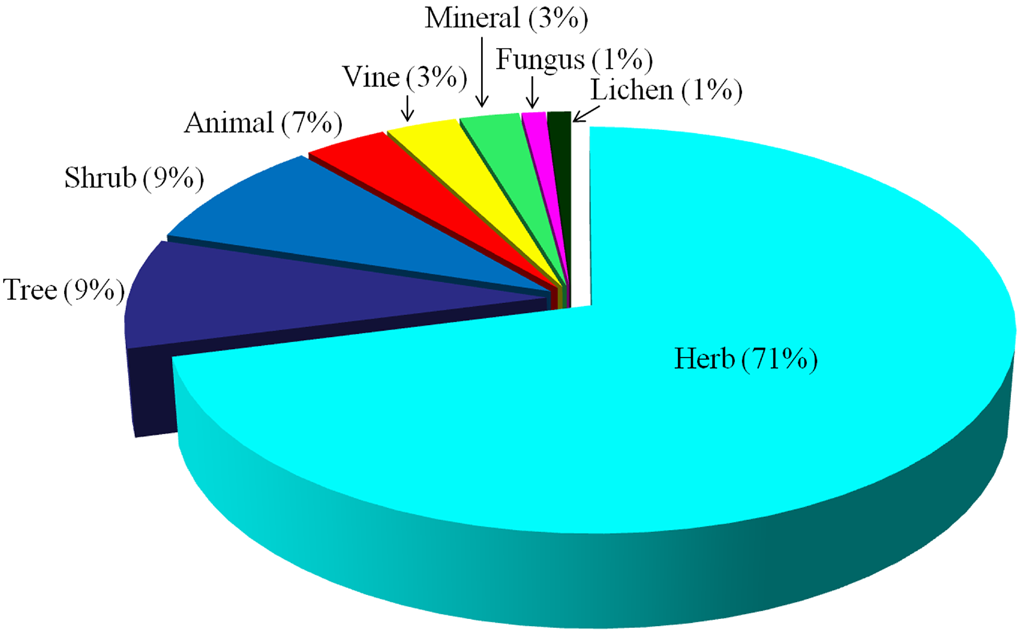


Supplementary Figure S2 Life forms of traditional Tibetan medicines used in the treatment of liver diseases.


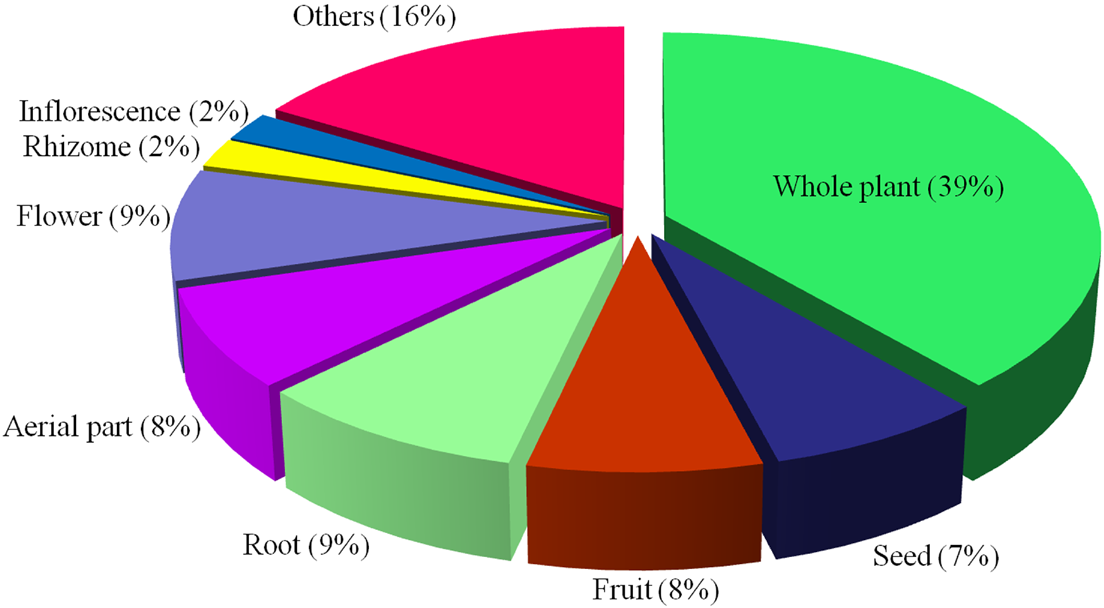


Supplementary Figure S3 Parts of traditional Tibetan medicines used in the treatment of liver diseases.

Supplementary Table S1 The related information of the 22 monographs referenced in our paper

| No. | Monograph | Author | The type of author | Ethnic group of author | Data origin of the monograph | Publication date of the monograph | Press of the monograph | Main content of the monograph | Language of the monograph | The type of the monograph | The number of species extracted from the monograph in our paper |
| --- | --- | --- | --- | --- | --- | --- | --- | --- | --- | --- | --- |
| 1 | Jing Zhu Materia Medica | Dimaer, D. Z. P. C. | Tibetan doctor | Tibetan | Ancient document and ethnobotanical survey | 2012 | Shanghai Science and Technology Press | Drug classification | Chinese version translated by maojizhu | Print edition | 33 |
| 2 | Dictionary of Chinese Ethnic Medicine | Jia, M. R. and Zhang, Y. | Botanist and scientist, respectively | Han Chinese | Data compilation | 2016 | China Medical Science and Technology Press | Drug compilation | Chinese | Print edition | 67 |
| 3 | Chinese Tibetan Materia Medica | Luo, D. S. | Scientist and botanist | Han Chinese | Ethnobotanical survey | 1997 | Ethnic Publishing Press | Drug introduction | Chinese | Print edition | 31 |
| 4 | Drug Standards of Tibetan Medicine | Chinese Pharmacopoeia Commission | National institution | — | Data compilation | 1995 | Ministry of Health of the People's Republic of China | Pharmacopeia | Chinese | Print edition | 20 |
| 5 | Chinese Tibetan Medicine (vol. 1-3) | Qinghai Institute for Drug Control | Local institution | — | Ethnobotanical survey | 1996 | Shanghai Science and Technology Press | Drug introduction | Chinese | Print edition | 15 |
| 6 | Chinese Herbalism for Tibetan Medicine | Editorial Board of Chinese Herbalism | National institution | — | Data compilation | 2002 | Shanghai Science and Technology Press | Drug compilation | Chinese | Print edition | 14 |
| 7 | Yunnan Province Annals (Medicine Annals) | Yunnan Editorial Board of Local Chronicles | Local institution | — | Data compilation | 1995 | Yunnan People’s Publishing Press | Drug compilation | Chinese | Print edition | 11 |
| 8 | Tibetan Medicine Annals | Yang, Y. C. | Scientist and botanist | Han Chinese | Ancient document and ethnobotanical survey | 1991 | Qinghai People's Publishing Press | Drug introduction | Chinese | Print edition | 9 |
| 9 | Commonly Used Tibetan Medicine Annals | Tian, S. Q. | Scientist | Han Chinese | Data compilation | 1997 | Sichuan Science and Technology Press | Drug compilation | Chinese | Print edition | 9 |
| 10 | Diqing Tibetan Medicine | Yang, J. S. and Chuchen, J. C. | Botanist and Tibetan doctor, respectively | Han Chinese and Tibetan, respectively | Ethnobotanical survey | 1987 | Nationalities Publishing Press of Yunnan | Drug introduction | Chinese | Print edition | 9 |
| 11 | Tibetan Medicine Standards | Health Bureau of Tibet, Qinghai, Sichuan, Gansu, Yunnan, and Xinjiang | Local institution | — | Data compilation | 1979 | Qinghai People’s Publishing Press | Pharmacopeia | Chinese | Print edition | 8 |
| 12 | Drug Illustration of Qinghai-Tibet Plateau (vol. 1-3) | Qinghai Institute of Plateau Biology | Local institution | — | Ethnobotanical survey | 1972, 1975 and 1978 | Qinghai People’s Publishing Press | Drug introduction | Chinese | Print edition | 7 |
| 13 | Chinese Ethnic Medicine Annals (vol. 1) | Editorial Board of Chinese Ethnic Medicine | Local institution | — | Data compilation | 1984 | People's Medical Publishing Press | Drug compilation | Chinese | Print edition | 6 |
| 14 | Chinese Ethnic Medicine Annals (vol. 2) | Editorial Board of Chinese Ethnic Medicine | Local institution | — | Data compilation | 1990 | People's Medical Publishing Press | Drug compilation | Chinese | Print edition | 4 |
| 15 | Chinese Ethnic Medicine Annals (vol. 3) | Editorial Board of Chinese Ethnic Medicine | Local institution | — | Data compilation | 2000 | Sichuan National Publishing Press | Drug compilation | Chinese | Print edition | 2 |
| 16 | Illustrated Handbook of Ethnic Medicine of Shangri-La | Yunnan University of TCM | University | — | Ethnobotanical survey | 2008 | Yunnan Science and Technology Press | Drug introduction | Chinese | Print edition | 2 |
| 17 | Medicinal Animals of Qinghai-Tibet Plateau | Ye, B. L. and Guo, P. J. | Scientist | Unknown | Ethnobotanical survey | 1998 | Shaanxi Science and Technology Press | Drug introduction | Chinese | Print edition | 1 |
| 18 | Annotation of Commonly Used Tibetan Medicine Prescription | Wu, H. C., Wang, M. S., and Dang, H. Q. | Scientist | Unknown | Data compilation | 2002 | Qinghai People's Publishing Press | Prescription compilation | Chinese | Print edition | — |
| 19 | Jing Jing Materia Medica | Gawu, D. J. | Scientist | Tibetan | Ethnobotanical survey | 1993 | Ethnic Publishing Press | Drug introduction | Tibetan | Print edition | 7 |
| 20 | Gannan Tibetan Medicine Flora of Qinghai-Tibet Plateau | Du, P. | Scientist | Han Chinese | Ethnobotanical survey | 2006 | Gansu Science and Technology Press | Drug introduction | Chinese | Print edition | 13 |
| 21 | Newly Revised Jing Zhu Materia Medica | Luo, D. S. | Scientist and botanist | Han Chinese | Ancient document and ethnobotanical survey | 2004 | Sichuan Science and Technology Press | Drug classification | Chinese | Print edition | 41 |
| 22 | Medicinal Minerals of Qinghai-Tibet Plateau | Xing, Z. G., Hu, X. M., Zhao, D. F., and Guo, P. J. | Scientist | Unknown | Data compilation | 1985 | Qinghai People’s Publishing Press | Drug compilation | Chinese | Print edition | 1 |

Supplementary Table S2 Natural Tibetan medicines for the treatment of liver diseases in traditional Tibetan medical system

| No. | Latin name | Tibetan name | Family | Life form | Used part | Treated liver diseases | Reported biological activities associated with liver diseases |
| --- | --- | --- | --- | --- | --- | --- | --- |
| 1 | *Acalypha australis* L. | Jia-xiao | Euphorbiaceae | Herb | Whole plant | Hepatitis (Du, 2006) | No report |
| 2 | *Aconitum naviculare* [(Brühl) Stapf](http://www.theplantlist.org/tpl1.1/record/kew-2619222) | Bang-ga | Ranunculaceae | Herb | Whole plant | Hepatitis and liver heat (Chinese Pharmacopoeia Commission, 1995; Tian, 1997) | No report |
| 3 | *Aconitum tanguticum* (Maxim.) Stapf | Bang-ga | Ranunculaceae | Herb | Whole plant | Hepatitis and liver heat (Chinese Pharmacopoeia Commission, 1995; Dimaer, 2012; Luo, 2004) | Inhibition of the proliferation of hepatocellular carcinoma cells HepG-2 (Fu, 2008) |
| 4 | *Aegypius monachus* Linnaeus | Qia-guo | Accipitridae | Animal | Bone | Liver ascites (Editorial Board of Chinese Ethnic Medicine, 1984) | No report |
|  |  |  |  |  | Bile | Liver diseases (Editorial Board of Chinese Ethnic Medicine, 1984; Luo, 1997) |  |
| 5 | *Agrimonia pilosa* Ledeb. | Zhe-ma | Rosaceae | Herb | Root or whole plant | Icterohepatitis (Jia and Zhang, 2016) | Inhibition of the proliferation of hepatocellular carcinoma cells HepG-2 (Song and Hao, 2011) |
| 6 | *Ajuga lupulina* Maxim. | Sheng-di | Labiatae | Herb | Whole plant | Toxic hepatic injury (Qinghai Institute of Plateau Biology, 1972, 1975 and 1978; Jia and Zhang, 2016) | No report |
| 7 | *Ajuga lupulina* Maxim. var. *major* Diels | Sheng-di | Labiatae | Herb | Whole plant | Toxic hepatic injury (Luo, 2004; Luo, 1997) | No report |
| 8 | Alum (KAl(SO_4_)_2_·12H_2_O) | Da-cuo-er | — | Mineral | — | Hepatitis (Xing et al., 1985) | No report |
| 9 | *Anemone obtusiloba* D.Don | Su-ga | Ranunculaceae | Herb | Aerial part, root or fruit | Hepatitis (Health Bureau of Tibet, Qinghai, Sichuan, Gansu, Yunnan, and Xinjiang, 1979) | No report |
| 10 | *Anemone rivularis* Buch. -Ham. [ex DC.](http://www.theplantlist.org/tpl1.1/record/kew-2638651) | Su-ga | Ranunculaceae | Herb | Aerial part, root or fruit | Hepatitis (Luo, 1997) | No report |
| 11 | *Artemisia demissa* Krasch. | Cha-er-wang-mu-bao | Compositae | Herb | Whole plant | Liver heat (Qinghai Institute for Drug Control, 1996) | No report |
| 12 | *Artemisia frigida* Willd. | Kan-jia-ga-bu | Compositae | Herb | Aerial part | Liver heat (Luo, 1997) | No report |
| 13 | *Artemisia hedinii* Ostenf. | Sang-zi-na-bo | Compositae | Herb | Aerial part | Acute icterohepatitis (Chinese Pharmacopoeia Commission, 1995; Jia and Zhang, 2016) | No report |
| 14 | *Artemisia scoparia* Waldst. [& Kitam.](http://www.theplantlist.org/tpl1.1/record/gcc-142288) | Cha-er-wang-ga-bao | Compositae | Herb | Whole plant | Icterohepatitis and liver heat (Qinghai Institute for Drug Control, 1996; Editorial Board of Chinese Ethnic Medicine, 2000) | Antioxidant and anti-inflammatory activities (Habib and Waheed, 2013; Singh et al., 2009) |
| 15 | *Berberis brachypoda* Maxim. | Ji-er-wa | Berberidaceae | Shrub | Root, stem or their endothelium | Icterohepatitis (Jia and Zhang, 2016) | No report |
| 16 | *Bidens bipinnata* L. | Qie-cai-man-ba | Compositae | Herb | Whole plant | Hepatitis (Luo, 2004) | Hepatoprotective effects against CCl_4_-induced acute liver injury and liver fibrosis (Zhong et al., 2007; Yuan et al., 2008) |
| 17 | *Bidens pilosa* L. | Qie-cai-man-ba | Compositae | Herb | Whole plant | Hepatitis (Dimaer, 2012; Luo, 2004) | No report |
| 18 | *Brassica rapa* L. | Xiong-ma | Cruciferae | Herb | Root-tuber | Toxic hepatopathy (Jia and Zhang, 2016) | No report |
| 19 | *Bupleurum chinense* DC. | Si-ri-a-sai-er-bao | Umbelliferae | Herb | Root | Hepatitis (Du, 2006) | Hepatoprotective effect and inhibition of the proliferation of hepatocellular carcinoma cells SMMC-7721 (Wang and Nan, 2008; Yang et al., 2012) |
| 20 | *Bupleurum marginatum* Wall. ex DC. | Si-la-ga-bao | Umbelliferae | Herb | Root | Hepatitis (Du, 2006) | Hepatoprotective effect (Du et al., 2014) |
| 21 | *Calendula officinalis* L. | Ge-gong-mai-duo | Compositae | Herb | Inflorescence | Liver diseases (Jia and Zhang, 2016) | No report |
| 22 | *Cardamine pratensis* L. | Ou-ba-en-bao | Cruciferae | Herb | Aerial part | Hepatitis (Jia and Zhang, 2016) | No report |
| 23 | *Cassia fistula* Linn. | Dong-ka | Leguminosae | Tree | Fruit | Hepatitis, toxic hepatopathy and liver heat (Chinese Pharmacopoeia Commission, 1995; Dimaer, 2012; Tian, 1997) | No report |
| 24 | *Catabrosa aquatica* (L.) [P. Beauv.](http://www.theplantlist.org/tpl1.1/record/kew-403714) | Dang-bu-ga-re | Gramineae | Herb | Whole plant | Hepatitis (Dimaer, 2012; Luo, 2004; Luo, 1997) | No report |
| 25 | *Chrysosplenium carnosum* Hook.f. & Thomson | Ya-ji-ma | Saxifragaceae | Herb | Whole plant | Acute icterohepatitis, liver cirrhosis and liver heat (Luo, 2004; Jia and Zhang, 2016) | No report |
| 26 | *Chrysosplenium davidianum* Decne. ex Maxim. | Ya-ji-ma | Saxifragaceae | Herb | Whole plant | Acute icterohepatitis (Yunnan Editorial Board of Local Chronicles, 1995) (Yang and Chuchen, 1987) | No report |
| 27 | *Chrysosplenium griffithii* Hook.f. [& Thomson](http://www.theplantlist.org/tpl1.1/record/kew-2720161) | Ya-ji-ma | Saxifragaceae | Herb | Whole plant | Acute icterohepatitis, liver cirrhosis and liver heat (Dimaer, 2012; Health Bureau of Tibet, Qinghai, Sichuan, Gansu, Yunnan, and Xinjiang, 1979; Yunnan Editorial Board of Local Chronicles, 1995) | No report |
| 28 | *Chrysosplenium lanuginosum* [Hook.f. & Thomson](http://www.theplantlist.org/tpl1.1/record/kew-2720185) | Ya-ji-ma | Saxifragaceae | Herb | Whole plant | Acute icterohepatitis and liver cirrhosis (Luo, 2004) | No report |
| 29 | *Chrysosplenium nepalense* [D.Don](http://www.theplantlist.org/tpl1.1/record/kew-2720212) | Ya-ji-ma | Saxifragaceae | Herb | Whole plant | Acute icterohepatitis and liver cirrhosis (Jia and Zhang, 2016; Yunnan Editorial Board of Local Chronicles, 1995) | No report |
| 30 | *Chrysosplenium nudicaule* [Bunge](http://www.theplantlist.org/tpl1.1/record/kew-2720220) | Ya-ji-ma | Saxifragaceae | Herb | Whole plant | Acute icterohepatitis, liver necrosis and liver cirrhosis (Dimaer, 2012; Editorial Board of Chinese Ethnic Medicine, 1990; Editorial Board of Chinese Herbalism, 2002) | No report |
| 31 | *Chrysosplenium uniflorum* Maxim. | Ya-ji-ma | Saxifragaceae | Herb | Whole plant | Acute icterohepatitis and hepatitis (Jia and Zhang, 2016) | No report |
| 32 | Cinnabar (HgS) | Jue-la | — | Mineral | — | Liver heat (Luo, 1997; Qinghai Institute for Drug Control, 1996) | No report |
| 33 | *Cinnamomum cassia* [(L.) J.Presl](http://www.theplantlist.org/tpl1.1/record/kew-2721201) | Xing-ca | Lauraceae | Tree | Bark | Liver diseases (Yunnan Editorial Board of Local Chronicles, 1995; Editorial Board of Chinese Herbalism, 2002) | No report |
| 34 | *Cinnamomum mairei* H.Lév. | Xing-ca | Lauraceae | Tree | Bark | Liver diseases (Jia and Zhang, 2016) | No report |
| 35 | *Cissampelopsis volubilis* [(Blume) Miq.](http://www.theplantlist.org/tpl1.1/record/gcc-16497) | Sai-bao-gu-zhui | Compositae | Herb | Whole plant | Icterohepatitis (Luo, 2004; Luo, 1997) | No report |
| 36 | *Clematis aethusifolia* Turcz. | Yi-meng-ga-bu | Ranunculaceae | Vine | Aerial part | Hepatic schistosomiasis (Luo, 1997) | No report |
| 37 | *Codonopsis convolvulacea* Kurz | Ni-wa | Campanulaceae | Herb | Flower | Liver heat (Editorial Board of Chinese Herbalism, 2002) | No report |
| 38 | *Comastoma pedunculatum* (Royle ex G. Don) Holub | Di-wa | Gentianaceae | Herb | Whole plant | Icterohepatitis (Du, 2006; Yang, 1991) | No report |
| 39 | *Comastoma pulmonarium* (Turcz.) Toyok. | Jia-di-jia-zha | Gentianaceae | Herb | Whole plant | Liver heat (Luo, 2004; Yang and Chuchen, 1987) | No report |
| 40 | *Commiphora myrrha* (Nees) Engl. | Ge-ge-le | Burseraceae | Tree | Resin | Liver diseases (Gawu, 1993) | Hepatoprotective activity (Ahmad et al., 2015) |
| 41 | *Coriolus versicolor* (L. ex Fr.) Quel. | Yun-zhi | Polyporaceae | Fungus | Fruiting body | Hepatitis B and chronic hepatitis (Yunnan University of TCM, 2008) | No report |
| 42 | *Corydalis adunca* Maxim. | Pa-meng-si-wa | Papaveraceae | Herb | Whole plant | Liver heat (Luo, 1997; Editorial Board of Chinese Ethnic Medicine, 1990) | No report |
| 43 | *Corydalis bungeana* Turcz. | Jia-zhou-si-wa | Papaveraceae | Herb | Whole plant | Chronic hepatitis and icterohepatitis (Luo, 1997; Jia and Zhang, 2016) | Hepatoprotective activity (Han et al., 2009) |
| 44 | *Corydalis curviflora* Maxim. [ex Hemsl.](http://www.theplantlist.org/tpl1.1/record/kew-2738624) | Qia-po-zi-zi-man-ba | Papaveraceae | Herb | Whole plant | Hepatitis (Luo, 1997) | No report |
| 45 | *Corydalis dasyptera* Maxim. | Ge-zhou-se-bu | Papaveraceae | Herb | Whole plant | Icterohepatitis (Dimaer, 2012; Jia and Zhang, 2016) | No report |
| 46 | *Corydalis delphinioides* Fedde | Pa-xia-ga-man-ba | Papaveraceae | Herb | Whole plant | Liver diseases (Luo, 1997; Yunnan University of TCM, 2008) | No report |
| 47 | *Corydalis nigroapiculata* C.Y.Wu | Gu-zhu-si-wa | Papaveraceae | Herb | Whole plant | Icterohepatitis (Editorial Board of Chinese Ethnic Medicine, 2000) | No report |
| 48 | *Corydalis pauciflora* [(Steph. ex Willd.) Pers.](http://www.theplantlist.org/tpl1.1/record/kew-2739088) | Ba-xia-wa | Papaveraceae | Herb | Whole plant | Hepatitis (Luo, 2004) | No report |
| 49 | *Corydalis scaberula* Maxim. | Dong-ri-si-ba | Papaveraceae | Herb | Whole plant | Hepatitis and liver heat (Chinese Pharmacopoeia Commission, 1995; Dimaer, 2012; Jia and Zhang, 2016) | No report |
| 50 | *Corydalis stricta* Steph. ex DC. | Pa-meng-si-wa | Papaveraceae | Herb | Whole plant | Hepatitis (Jia and Zhang, 2016) | No report |
| 51 | *Corydalis taliensis* Franch. | Ba-ri-ba-da-man-ba | Papaveraceae | Herb | Whole plant | Hepatitis (Luo, 1997) | No report |
| 52 | *Corydalis tangutica* Peschkova | Qia-po-zi-zi-ma-ba | Papaveraceae | Herb | Whole plant or root | Hepatitis (Luo, 2004; Jia and Zhang, 2016) | No report |
| 53 | *Corydalis tsayulensis* C.Y.Wu & H.Chuang | Zha-sang | Papaveraceae | Herb | Whole plant or root | Hepatitis (Jia and Zhang, 2016; Editorial Board of Chinese Herbalism, 2002) | No report |
| 54 | *Crepis lignea* (Vaniot) Babc. | Za-chi | Compositae | Herb | Root | Icterohepatitis (Luo, 2004) | No report |
| 55 | *Cuscuta australis* R.Br. | Zhu-xia-ba | Convolvulaceae | Herb | Seed or Whole plant | Hepatitis (Du, 2006) | No report |
| 56 | *Cuscuta chinensis* Lam. | Niu-xia-qiong-wa | Convolvulaceae | Herb | Whole plant | Icterohepatitis (Dimaer, 2012; Jia and Zhang, 2016) | Hepatoprotective and antioxidant effects (Yen et al., 2007) |
| 57 | *Cynanchum forrestii* Schltr. | E-du-mu-niu | Asclepiadaceae | Herb | Whole plant | Hepatitis (Yang and Chuchen, 1987) | No report |
| 58 | *Dalbergia odorifera* T.C. Chen | Zhan-tan-ma-bao | Leguminosae | Tree | Heartwood | Liver heat (Yang, 1991) | No report |
| 59 | *Delphinium batangense* Finet & Gagnep. | Xia-gang-ba | Ranunculaceae | Herb | Aerial part | Liver heat (Jia and Zhang, 2016) | No report |
| 60 | *Dracocephalum calophyllum* Hand.-Mazz. | Yao-qing-lan | Labiatae | Herb | Whole plant or Aerial part | Hepatitis (Luo, 2004; Yunnan Editorial Board of Local Chronicles, 1995) | No report |
| 61 | *Dracocephalum heterophyllum* Benth. | Ji-zi-qing-bao | Labiatae | Herb | Aerial part | Icterohepatitis (Chinese Pharmacopoeia Commission, 1995; Tian, 1997) | Protective effects against hypoxia-induced liver injury and ConA-induced acute hepatitis (Ma et al., 1995; Zheng et al., 2016) |
| 62 | *Dubyaea atropurpurea* (Franch.) Stebbins | Za-chi-na-bo | Compositae | Herb | Whole plant or inflorescence | Hepatitis (Yunnan Editorial Board of Local Chronicles, 1995) | No report |
| 63 | *Entada phaseoloides* (L.) Merr. | Qing-ba-xiao-xia | Leguminosae | Vine | Seed | Liver heat and toxic hepatopathy (Chinese Pharmacopoeia Commission, 1995; Dimaer, 2012; Tian, 1997) | No report |
| 64 | *Ephedra equisetina* [Bunge](http://www.theplantlist.org/tpl1.1/record/kew-332918) | Cai-dun-mu | Ephedraceae | Shrub | Aerial part | Liver heat (Dimaer, 2012; Luo, 1997; Qinghai Institute for Drug Control, 1996) | No report |
| 65 | *Erodium stephanianum* Willd. | Xin-tuo-li-ga-bao | Geraniaceae | Herb | Whole plant | Hepatitis (Du, 2006) | No report |
| 66 | *Fallopia aubertii* (L.Henry) Holub | Le-zhe | Polygonaceae | Shrub | Stem | Liver heat (Qinghai Institute of Plateau Biology, 1972, 1975 and 1978; Qinghai Institute for Drug Control, 1996) | No report |
| 67 | Ferrum | Jiu-tai | — | Mineral | — | Toxic hepatopathy (Qinghai Institute for Drug Control, 1996; Yang and Chuchen, 1987) | No report |
| 68 | *Fraxinus sikkimensis* (Lingelsh.) Hand.-Mazz. | Da-bu-sang | Oleaceae | Tree | Bark | Liver heat (Luo, 2004; Yunnan Editorial Board of Local Chronicles, 1995) | No report |
| 69 | *Ganoderma sinense* Zhao. Xu et Zhang | Guo-xia-mo-bu | Polyporaceae | Fungus | Fruiting body | Hepatitis (Luo, 1997) | No report |
| 70 | *Gentiana crassicaulis* Duthie ex Burkill | Jie-ji-na-bao | Gentianaceae | Herb | Flower | Hepatitis and liver heat (Dimaer, 2012; Health Bureau of Tibet, Qinghai, Sichuan, Gansu, Yunnan, and Xinjiang, 1979; Jia and Zhang, 2016) | No report |
| 71 | *Gentiana macrophylla* Pall. | Jie-ji-na-bao | Gentianaceae | Herb | Flower or whole plant | Hepatitis (Dimaer, 2012; Jia and Zhang, 2016) | Protective effects against alcohol and CCl_4_-induced liver injury (Kang et al., 2012; Zhang et al., 2014) |
| 72 | *Gentiana waltonii* Burkill | Jie-ji-na-bao | Gentianaceae | Herb | Root | Icterohepatitis (Luo, 2004; Jia and Zhang, 2016) | No report |
| 73 | *Gentiana lhassica* Burkill | Jie-ji-na-bao | Gentianaceae | Herb | Whole plant | Hepatitis (Luo, 2004) | No report |
| 74 | *Gentiana futtereri* Diels & Gilg | Jie-ji-mo-bao | Gentianaceae | Herb | Flower | Hepatitis (Dimaer, 2012; Luo, 2004; Jia and Zhang, 2016) | Protective effect against CCl_4_-induced liver injury (Tang, 2011) |
| 75 | *Gentiana obconica* T.N.Ho | Jie-ji-mo-bao | Gentianaceae | Herb | Flower | Hepatitis (Luo, 2004; Jia and Zhang, 2016) | No report |
| 76 | *Gentiana erectosepala* T.N.Ho | Jie-ji-en-bao | Gentianaceae | Herb | Flower | Hepatitis (Luo, 2004; Jia and Zhang, 2016) | No report |
| 77 | *Gentiana rhodantha* Franch. ex Hemsl. | Jie-ji-ma-bao | Gentianaceae | Herb | Whole plant | Liver heat and icterohepatitis (Jia and Zhang, 2016) | No report |
| 78 | *Gentiana robusta* King ex Hook.f. | Jie-ji-ga-bao | Gentianaceae | Herb | Root | Icterohepatitis (Luo, 2004; Yang and Chuchen, 1987) | No report |
| 79 | *Gentiana straminea* Maxim. | Jie-ji-ga-bao | Gentianaceae | Herb | Flower | Hepatitis (Chinese Pharmacopoeia Commission, 1995; Dimaer, 2012) | No report |
| 80 | *Gentiana tibetica* King ex Hook.f. | Jie-ji-ga-bao | Gentianaceae | Herb | Whole plant or flower | Icterohepatitis (Dimaer, 2012; Jia and Zhang, 2016; Yang, 1991) | No report |
| 81 | *Gentiana nubigena* Edgew. | Bang-jian | Gentianaceae | Herb | Flower | Hepatitis (Du, 2006; Health Bureau of Tibet, Qinghai, Sichuan, Gansu, Yunnan, and Xinjiang, 1979) | No report |
| 82 | *Gentiana purdomii* C.Marquand | Bang-jian | Gentianaceae | Herb | Flower | Hepatitis (Health Bureau of Tibet, Qinghai, Sichuan, Gansu, Yunnan, and Xinjiang, 1979; Jia and Zhang, 2016) | No report |
| 83 | *Gentiana szechenyii* Kanitz | Bang-jian | Gentianaceae | Herb | Flower | Hepatitis (Health Bureau of Tibet, Qinghai, Sichuan, Gansu, Yunnan, and Xinjiang, 1979; Jia and Zhang, 2016) | No report |
| 84 | *Gentiana algida* Pall. | Bang-jian | Gentianaceae | Herb | Whole plant | Hepatitis (Jia and Zhang, 2016) | No report |
| 85 | *Gentiana algida* Pall. var. *przewalskii* (Maxim.) Kusn. | Bang-jian | Gentianaceae | Herb | Flower | Hepatitis (Jia and Zhang, 2016) | No report |
| 86 | *Gentianopsis barbata* (Froel.) Ma | Jia-di | Gentianaceae | Herb | Whole plant | Icterohepatitis, liver heat and hepatitis (Luo, 2004; Jia and Zhang, 2016) | No report |
| 87 | *Gentianopsis grandis* (Harry Sm.) Ma | Jia-di | Gentianaceae | Herb | Whole plant | Hepatitis (Luo, 2004; Jia and Zhang, 2016) | No report |
| 88 | *Gentianopsis paludosa* (Hook.f.) Ma | Jia-di | Gentianaceae | Herb | Whole plant | Icterohepatitis and hepatitis (Chinese Pharmacopoeia Commission, 1995; Dimaer, 2012; Jia and Zhang, 2016) | Protective effect against CCl_4_-induced liver injury (Ren et al., 2008) |
| 89 | *Geum aleppicum* Jacq. | Za-e-bao | Rosaceae | Herb | Whole plant | Hepatitis (Luo, 2004; Luo, 1997) | No report |
| 90 | *Gossampinus malabarica* Merr. | Na-ka-bu-sa | Bombacaceae | Tree | Flower | Liver heat (Chinese Pharmacopoeia Commission, 1995; Dimaer, 2012; Gawu, 1993) | Protective effects on immunological liver injury and liver fibrosis (Wu et al., 2012; Tang et al., 2014) |
| 91 | *Halenia corniculata* (L.) Cornaz | Jia-di-ran-guo | Gentianaceae | Herb | Aerial part | Acute icterohepatitis and hepatitis B (Jia and Zhang, 2016) | Protective effect against D-galactosamine-induced liver injury (Gui et al., 2015) |
| 92 | *Herminium alaschanicum* Maxim. | Wang-la-man-ba | Orchidaceae | Herb | Tuber | Chronic hepatitis (Luo, 2004) | No report |
| 93 | *Hippophae rhamnoides* L. | Da-bu | Elaeagnaceae | Shrub or tree | Fruit | Liver diseases (Dimaer, 2012; Jia and Zhang, 2016) | Hepatoprotective and antioxidant effects (Geetha et al., 2008; Ting et al., 2011), and antiproliferative activity on human liver cancer cell lines HepG-2 (Grey et al., 2010) |
| 94 | *Hippuris vulgaris* L. | Dong-bu-ga-la | Hippuridaceae | Herb | Whole plant or rhizome | Hepatitis (Dimaer, 2012; Qinghai Institute of Plateau Biology, 1972, 1975 and 1978) | No report |
| 95 | *Holarrhena antidysenterica* [(Roth) Wall. ex A.DC.](http://www.theplantlist.org/tpl1.1/record/kew-464913) | Du-mo-niu | Apocynaceae | Tree | Seed | Liver diseases (Chinese Pharmacopoeia Commission, 1995; Tian, 1997) | No report |
| 96 | *Hypericum bellum* [H.L. Li](http://www.theplantlist.org/tpl1.1/record/kew-2857799) | Jia-xiang-wang-qiu | Guttiferae | Shrub | Fruit | Hepatitis (Luo, 2004; Yunnan Editorial Board of Local Chronicles, 1995) | No report |
| 97 | *Inula helenium* L. | Ma-nu-ba-zha | Compositae | Herb | Root | Liver diseases (Jia and Zhang, 2016) | No report |
| 98 | *Iris ensata* Thunb. | Zhe-ze | Iridaceae | Herb | Seed | Icterohepatitis (Jia and Zhang, 2016) | No report |
| 99 | *Iris lactea* Pall. var. *chinensis* (Fisch.) Koidz. | Mu-zhe | Iridaceae | Herb | Seed | Icterohepatitis (Jia and Zhang, 2016) | No report |
| 100 | *Ixeris chinensis* [(Thunb. ex Thunb.) Nakai](http://www.theplantlist.org/tpl1.1/record/gcc-108473) | Za-chi | Compositae | Herb | Whole plant | Icterohepatitis (Luo, 2004) | Hepatoprotective effect (Lin et al., 1994) |
| 101 | *Juniperus formosana* Hayata | Xiu-ba-cai-jian | Cupressaceae | Tree | Fruit | Liver diseases (Luo, 1997) | No report |
| 102 | *Lagopsis supina* [(Steph. ex Willd.) Ikonn.-Gal.](http://www.theplantlist.org/tpl1.1/record/kew-107319) | Xing-tuo-li-ga-bao | Labiatae | Herb | Aerial part | Liver heat (Yunnan Editorial Board of Local Chronicles, 1995) | No report |
| 103 | *Lamium amplexicaule* L. | Zha-xi-ge-ze | Labiatae | Herb | Whole plant | Icterohepatitis (Du, 2006; Yunnan Editorial Board of Local Chronicles, 1995) | No report |
| 104 | *Lancea tibetica* Hook. f. & Thomson | Wa-ya-he-ba | Scrophulariaceae | Herb | Whole plant | Hepatitis (Jia and Zhang, 2016) | No report |
| 105 | Limonite | Jia-he-ji-duo | — | Mineral | — | Toxic hepatopathy (Gawu, 1993) | No report |
| 106 | *Lithospermum erythrorhizon* Siebold & Zucc. | Zhi-mao-he | Boraginaceae | Herb | Root | Hepatitis (Du, 2006) | Protective effect against CCl_4_-induced liver injury (Feng et al., 2010) |
| 107 | *Lomatogonium micranthum* Harry Sm. | Ji-he-di | Gentianaceae | Herb | Whole plant | Acute icterohepatitis (Du, 2006; Yang, 1991) | No report |
| 108 | *Lycopodium japonicum* Thunb. | Qu-sen-de-mo | Lycopodiaceae | Herb | Whole plant | Hepatitis (Luo, 1997) | No report |
| 109 | *Nigella damascena* L. | Si-re-na-bu | Ranunculaceae | Herb | Seed | Hepatitis, hepatomegaly and hepatic schistosomiasis (Luo, 1997; Qinghai Institute for Drug Control, 1996) | Protective effect against alcohol-induced liver injury (Yu et al., 2016) |
| 110 | *Orchis latifolia* L. var. *angustata* Maxim. | Wang-la | Orchidaceae | Herb | Tuber | Chronic hepatitis (Luo, 1997; Qinghai Institute of Plateau Biology, 1972, 1975 and 1978) | No report |
| 111 | *Oroxylum indicum* (L.) Kurz | Zhan-ba-ga | Bignoniaceae | Tree | Seed | Hepatitis (Dimaer, 2012; Qinghai Institute for Drug Control, 1996; Gawu, 1993) | No report |
| 112 | *Pedicularis cranolopha* Maxim. | Lu-ru-se-bao | Scrophulariaceae | Herb | Flower | Liver heat and hepatitis (Jia and Zhang, 2016; Gawu, 1993) | No report |
| 113 | *Pedicularis dichotoma* Bonati | Ji-zi-ma-bao | Scrophulariaceae | Herb | Whole plant | Hepatitis (Jia and Zhang, 2016) | No report |
| 114 | *Phyllophyton tibeticum* (Jacq ex Benth.) C.Y. Wu | Nian-du-ba | Labiatae | Herb | Whole plant | Hepatitis and toxic hepatic injury (Health Bureau of Tibet, Qinghai, Sichuan, Gansu, Yunnan, and Xinjiang, 1979) | No report |
| 115 | *Picrorhiza scrophulariiflora* Pennell | Hong-lian-mo-bu | Scrophulariaceae | Herb | Rhizome | Liver heat (Yang and Chuchen, 1987; Editorial Board of Chinese Herbalism, 2002) | Hepatoprotective, immunomodulatory and anti-inflammatory activities (Smit et al., 2000; Liu et al., 2002) |
| 116 | *Polygonum sinomontanum* Sam. | Ran-bu-ka-tu | Polygonaceae | Herb | Whole plant or rhizome | Liver heat (Qinghai Institute for Drug Control, 1996) | No report |
| 117 | *Populus cathayana* Rehder | Ma-geng | Salicaceae | Tree | Branch or leaf | Hepatitis (Luo, 2004) | No report |
| 118 | *Prunella hispida* Benth. | Xia-gu-cuo | Labiatae | Herb | Seed | Hepatitis and liver heat (Jia and Zhang, 2016) | No report |
| 119 | *Pseudolysimachion longifolium* (L.) Opiz | Wa-xia-ga | Scrophulariaceae | Herb | Whole plant | Hepatitis (Jia and Zhang, 2016) | No report |
| 120 | *Pyrethrum tatsienense* [(Bureau & Franch.) Ling ex C.Shih](http://www.theplantlist.org/tpl1.1/record/gcc-101899) | A-xia-sai-er-jun | Compositae | Herb | Whole plant or inflorescence | Hepatitis (Jia and Zhang, 2016) | Protective effect on acute hepatic injury induced by D-galactosamine (Lin et al., 2011) |
| 121 | *Rheum officinale* Baill. | Jun-zha | Polygonaceae | Herb | Root or fruit | Acute viral hepatitis (Jia and Zhang, 2016) | No report |
| 122 | *Rheum palmatum* L. | Jun-zha | Polygonaceae | Herb | Root or fruit | Liver diseases and acute viral hepatitis (Jia and Zhang, 2016; Editorial Board of Chinese Herbalism, 2002) | Antiviral effects against duck hepatitis B virus (Chung et al., 1997) |
| 123 | *Rhododendron anthopogon* D. Don | Ta-li-ga-bao | Ericaceae | Shrub | Leaf | Liver cancer and hepatomegaly (Luo, 2004) | No report |
| 124 | *Rhododendron anthopogonoides* Maxim. | Da-li | Ericaceae | Shrub | Flower and branch | Liver cancer and hepatomegaly (Qinghai Institute of Plateau Biology, 1972, 1975 and 1978) | No report |
| 125 | *Rhododendron primuliflorum* [Bureau & Franch.](http://www.theplantlist.org/tpl1.1/record/tro-12305045) | Da-li | Ericaceae | Shrub | Flower and branch | Liver cancer and hepatomegaly (Jia and Zhang, 2016) | No report |
| 126 | *Ribes alpestre* Wall. ex Decne. | Sai-guo | Saxifragaceae | Shrub | Fruit | Hepatitis (Luo, 2004) | No report |
| 127 | *Ribes himalense* Royle ex Decne. | Sai-guo | Saxifragaceae | Shrub | Fruit or stem endothelium | Hepatitis (Jia and Zhang, 2016; Editorial Board of Chinese Herbalism, 2002) | No report |
| 128 | *Rosa bella* [Rehder & E.H.Wilson](http://www.theplantlist.org/tpl1.1/record/rjp-11843) | Sai-guo | Rosaceae | Shrub | Fruit | Hepatitis (Luo, 2004; Jia and Zhang, 2016) | No report |
| 129 | *Rumex acetosa* L. | Xiao-mang | Polygonaceae | Herb | Root or rhizome | Hepatitis (Luo, 1997) | No report |
| 130 | *Salix microstachya* var. *bordensis* [(Nakai) C.F. Fang](http://www.theplantlist.org/tpl1.1/record/tro-28301656) | Jiang-ma | Salicaceae | Shrub | Root, stem or their endothelium | Icterohepatitis (Luo, 2004) | No report |
| 131 | *Salsola collina* Pall. | Da-cai-er | Chenopodiaceae | Herb | Aerial part | Liver heat (Luo, 1997) | No report |
| 132 | *Salvia bifidocalyx* C.Y.Wu & Y.C.Huang | Huang-hua-shu-wei-cao | Labiatae | Herb | Flower | Hepatitis (Yang and Chuchen, 1987) | No report |
| 133 | *Salvia flava* Forrest ex Diels | Ji-zi-se-bao | Labiatae | Herb | Root | Hepatitis (Jia and Zhang, 2016) | No report |
| 134 | *Sambucus chinensis* Lindl. | Yu-gou-xiang-na-bao | Caprifoliaceae | Herb | Whole plant | Liver heat (Luo, 2004) | Protective effect on acute hepatic injury induced by D-galactosamine, CCl_4_ or ConA (Yang et al., 2006) |
| 135 | *Saussurea arenaria* Maxim. | Za-chi-wa-mao-ka | Compositae | Herb | Whole plant | Hepatitis (Qinghai Institute of Plateau Biology, 1972, 1975 and 1978) | No report |
| 136 | *Saussurea brunneopilosa* Hand.-Mazz. | Za-chi-ba-mo-ka | Compositae | Herb | Aerial part | Hepatitis (Chinese Pharmacopoeia Commission, 1995; Editorial Board of Chinese Herbalism, 2002) | No report |
| 137 | *Saussurea graminea* Dunn | Za-chi-ba-mao-ka | Compositae | Herb | Aerial part or inflorescence | Hepatitis (Chinese Pharmacopoeia Commission, 1995; Luo, 1997) | No report |
| 138 | *Sedum multicaule* Wall. ex Lindl. | Can-a-wu-zi | Crassulaceae | Herb | Whole plant | Hepatitis (Luo, 1997) | No report |
| 139 | *Senecio scandens* Buch.-Ham. ex D.Don | Sai-bao-gu-zhui | Compositae | Herb | Whole plant | Icterohepatitis (Luo, 2004) | Hepatoprotective, antioxidant, antiviral, and anti-inflammatory effects (Wang et al., 2013) |
| 140 | *Skimmia multinervia* C.C. Huang | Xie-kan | Rutaceae | Tree | Leaf | Liver heat (Gawu, 1993) | No report |
| 141 | *Sonchus arvensis* L. | Zha-chi-na-bo | Compositae | Herb | Whole plant | Icterohepatitis and liver diseases (Luo, 2004; Jia and Zhang, 2016) | Hepatoprotective effect (Alkreathy et al., 2014) |
| 142 | *Sophora alopecuroides* L. | Ji-wa | Leguminosae | Herb | Seed | Icterohepatitis (Luo, 2004) | Hepatoprotective effect (Yang et al., 2008) |
| 143 | *Sophora glauca* var. *albescens* Rehder | Ji-wa | Leguminosae | Tree | Seed | Icterohepatitis (Jia and Zhang, 2016) | No report |
| 144 | *Sophora davidii* (Franch.) [Pavol.](http://www.theplantlist.org/tpl1.1/record/ild-7167) | Jie-bei-zhai-bu | Leguminosae | Shrub | Seed | Icterohepatitis (Luo, 2004; Yang and Chuchen, 1987) | No report |
| 145 | *Sphaerophysa salsula* (Pall.) DC. | La-wa-sa-ma | Leguminosae | Shrub | Root or fruit | Chronic hepatitis and liver cirrhosis (Du, 2006) | No report |
| 146 | *Stachys sieboldii* Miq. | Dong-na-duan-chi | Labiatae | Herb | Whole plant | Hepatitis (Luo, 2004; Yang, 1991) | No report |
| 147 | *Strophanthus divaricatus* (Lour.) Hoo[k. & Arn.](http://www.theplantlist.org/tpl1.1/record/kew-198213) | Tu-mu-rong | Apocynaceae | Shrub | Seed | Liver heat (Dimaer, 2012; Jia and Zhang, 2016) | No report |
| 148 | *Styrax macrocarpus* Cheng | Ge-ge-le | Styracaceae | Tree | Resin | Liver diseases (Luo, 1997) | No report |
| 149 | *Sus scrofa domestica* Brisson | Pa-cha | Suidae | Animal | Blood | Toxic hepatic injure (Health Bureau of Tibet, Qinghai, Sichuan, Gansu, Yunnan, and Xinjiang, 1979; Qinghai Institute for Drug Control, 1996) | No report |
| 150 | *Synotis erythropappa* (Bureau & Franch.) C. Jeffrey & Y.L. Chen | Yu-gu-xing | Compositae | Herb | Whole plant | Liver heat (Luo, 2004) | No report |
| 151 | *Taraxacum eriopodum* (D.Don) DC. | Ku-mang | Compositae | Herb | Whole plant | Liver diseases (Jia and Zhang, 2016) | No report |
| 152 | *Taraxacum lugubre* Dahlst. | Ke-er-mang | Compositae | Herb | Whole plant | Hepatitis (Du, 2006) | No report |
| 153 | *Tephroseris rufa* [(Hand.-Mazz.) B.Nord.](http://www.theplantlist.org/tpl1.1/record/gcc-30118) | Sai-bao-gu-zhui | Compositae | Herb | Whole plant | Icterohepatitis (Luo, 2004; Yang, 1991) | No report |
| 154 | *Thalictrum acutifolium* (Hand. -Mazz.) B. Boivin | Si-la-na-bu-man-ba | Ranunculaceae | Herb | Inflorescence or fruit | Hepatitis, hepatomegaly and hepatic schistosomiasis (Luo, 1997; Qinghai Institute of Plateau Biology, 1972, 1975 and 1978) | No report |
| 155 | *Thalictrum foliolosum* DC. | Gong-bu-e-zheng | Ranunculaceae | Herb | Root or rhizome | Viral hepatitis (Luo, 2004; Luo, 1997) | No report |
| 156 | *Thalictrum rutifolium* Hook. f. & Thomson | E-zhen | Ranunculaceae | Herb | Flower or fruit | Viral hepatitis, hepatomegaly and hepatic schistosomiasis (Du, 2006) | No report |
| 157 | *Thamnolia subuliformis* (Ehrh.) Culb. | Sai-er-gu | Icmadophilaceae | Lichen | Whole body | Hepatitis and liver heat (Editorial Board of Chinese Herbalism, 2002; Yang, 1991) | No report |
| 158 | *Thladiantha setispina* A.M. Lu & Zhi Y. Zhang | Sai-ji-mei-duo | Cucurbitaceae | Vine | Seed | Liver heat and icterohepatitis (Yang, 1991) | No report |
| 159 | *Thlaspi arvense* L. | Zhai-ka | Cruciferae | Herb | Seed | Liver diseases (Editorial Board of Chinese Herbalism, 2002) | No report |
| 160 | *Tinospora capillipes* Gagnep. | Le-zhe | Menispermaceae | Vine | Rattan cane | Hepatitis and liver heat (Chinese Pharmacopoeia Commission, 1995; Editorial Board of Chinese Ethnic Medicine, 1990) | No report |
| 161 | *Tongoloa dunnii* (H. Boissieu) H. Wolff | Si-la-ga-bao | Umbelliferae | Herb | Fruit | Hepatitis (Jia and Zhang, 2016) | No report |
| 162 | *Trichosanthes lepiniana* (Naudin) Cogn. | Se-ji-mei-duo | Cucurbitaceae | Vine | Seed | Icterohepatitis (Luo, 2004) | No report |
| 163 | Trona | Pu-duo | — | Mineral | — | Toxic hepatopathy (Tian, 1997; Editorial Board of Chinese Herbalism, 2002) | No report |
| 164 | *Usnea diffracta* Vain. | Ou-guai | Usneaceae | Lichen | Whole body | Liver heat (Gawu, 1993) | No report |
| 165 | *Verbena officinalis* L. | Ma-ben-cou | Verbenaceae | Herb | Whole plant | Hepatitis (Editorial Board of Chinese Ethnic Medicine, 1984; Qinghai Institute for Drug Control, 1996) | Hepatoprotective and anti-inflammatory effects (Deepak and Handa, 2000; Yu, 2013) |
| 166 | *Vicia cracca* L. | Xi-wu-sai | Leguminosae | Herb | Whole plant | Icterohepatitis (Jia and Zhang, 2016) | No report |
